# Pathways to clinical CLARITY: volumetric analysis of irregular, soft, and heterogeneous tissues in development and disease

**DOI:** 10.1038/s41598-017-05614-4

**Published:** 2017-07-19

**Authors:** Brian Hsueh, Vanessa M. Burns, Philip Pauerstein, Katherine Holzem, Li Ye, Kristin Engberg, Ai-Chi Wang, Xueying Gu, Harini Chakravarthy, H. Efsun Arda, Gregory Charville, Hannes Vogel, Igor R. Efimov, Seung Kim, Karl Deisseroth

**Affiliations:** 1 Department of Bioengineering, Stanford University, Stanford, CA 94305 USA; 2 Department of Chemical and Systems Biology, Stanford University, Stanford, CA 94305 USA; 3 Department of Developmental Biology, Stanford University, Stanford, CA 94305 USA; 4 Department of Biomedical Engineering, Washington University in St. Louis, St. Louis, MO 63130 USA; 5 Department of Psychiatry and Behavioral Sciences, Stanford University, Stanford, CA 94305 USA; 6 Howard Hughes Medical Institute, Stanford, CA 94305 USA; 7 Department of Pathology, Stanford University, Stanford, CA 94305 USA; 8 Department of Biomedical Engineering, The George Washington University, Washington, DC 20052 USA

## Abstract

Three-dimensional tissue-structural relationships are not well captured by typical thin-section histology, posing challenges for the study of tissue physiology and pathology. Moreover, while recent progress has been made with intact methods for clearing, labeling, and imaging whole organs such as the mature brain, these approaches are generally unsuitable for soft, irregular, and heterogeneous tissues that account for the vast majority of clinical samples and biopsies. Here we develop a biphasic hydrogel methodology, which along with automated analysis, provides for high-throughput quantitative volumetric interrogation of spatially-irregular and friable tissue structures. We validate and apply this approach in the examination of a variety of developing and diseased tissues, with specific focus on the dynamics of normal and pathological pancreatic innervation and development, including in clinical samples. Quantitative advantages of the intact-tissue approach were demonstrated compared to conventional thin-section histology, pointing to broad applications in both research and clinical settings.

## Introduction

In both research and clinical settings, established techniques for visualizing biological samples enable either high-resolution imaging through thin sections or large scale volumetric imaging, but not both. Standard histopathology facilitates high-resolution anatomical and molecular phenotyping, but is limited to thin sections insufficient for directly observing complex three-dimensional features, such as ductal topology, tumor boundaries, or long-range neuronal projections, which may have scientific or diagnostic implications. Coupled with the numerous practical limitations of conventional histology^1^ related to laborious and imprecise sample sectioning, reconstruction, and quantification, investigation of cellular or subcellular structures has largely been limited to two-dimensional features visualized in a small number of representative samples. On the other hand, volumetric imaging techniques such as optical coherence tomography enable organ-wide analysis of three-dimensional structures, but sacrifice spatial resolution and molecular information, losing detailed features at the cellular level^2^.

For example, this tradeoff is severely limiting in the peripheral and enteric nervous systems, which play critical roles in development, mature physiology, and diseases including diabetic neuropathy and gastroparesis^3^. There exists a pressing need for quantification of corresponding fine volumetric features in a variety of clinical scenarios (e.g., measuring intra-epidermal nerve fiber topology and density in diabetic neuropathy or other pain disorders is increasingly understood to be of research and clinical value for both biomarker development and as a target endpoint for identifying and validating new treatments^4, 5^). With similar clinical motivations, diverse attempts have been made to characterize pancreatic architecture using either organ-wide analysis of macro-structural components^6^ or high-resolution analysis of small numbers of individual islet cross-sections^7^. However, simultaneous whole-organ and cellular-level analysis has remained elusive, particularly with regard to human specimens.

To achieve practical and quantitative high-resolution analysis of these heterogeneous and distributed systems, new methods for observing both macro- and micro-structural features were needed. While development of numerous clearing methodologies, including CLARITY, iDISCO, CUBIC, uDISCO, and SWITCH^8–12^, has shown that solid mouse tissues such as brain, lung, heart, and kidney, can be cleared and labelled, several key challenges remain: these methods are not widely suitable for soft, fragile, and irregular tissue targets such as those commonly found in clinical settings; material changes to the tissue rendered them incompatible with existing clinical analysis; and the use of specialized (and in some cases corrosive) chemical tools or customized devices represent practical barriers to adoption in clinical settings.

Among available tissue-clearing methods (reviewed in detail elsewhere^13, 14^), several considerations led us to optimize CLARITY in this direction and to enhance this platform for a broad array of clinical and research applications. First, CLARITY is uniquely compatible with detection and quantification of many categories of widely-used molecular labels with diagnostic utility, including nucleic acid probes^15^. Second, CLARITY permits multiple rounds of staining in large volumes of tissue^8, 16^, which is useful for multiplexing beyond the limits of spectral separation and is particularly important for rare or precious specimens, such as human banked or biopsied tissue. Equally critical for clinical applications, CLARITY is directly compatible with specimens preserved using common clinical fixation techniques, including formalin and flash freezing, avoiding challenges introduced by the use of innovative but non-standard tissue fixation methods. Finally, CLARITY preserves endogenous fluorescence; in contrast, organic solvent-based methods quickly quench native signals, which is relevant given the numerous multi-color fluorescent reporter tools used in research settings^17, 18^. However CLARITY has not yet been developed for heterogeneous and irregular tissues as encountered clinically.

Here we develop a biphasic hydrogel approach, involving no specialized expertise, custom equipment, or expensive reagents, to enable utilization of 3D histology in the standard research or clinical lab workflow. We highlight opportunities for three-dimensional discovery by using the approach to analyze the development of pancreatic innervation in mouse and human, quantitatively describing multiple phases of neural remodeling in developing pancreatic islets in an automated, high-throughput manner across whole mouse and human organs. Biphasic hydrogels and the pipeline of practical chemical, optical, and computational tools demonstrate an approach with potentially widespread research and clinical applicability.

## Results

### Biphasic CLARITY composites: hydrogel chemistry for irregular or fragile samples

**Figure 1:**
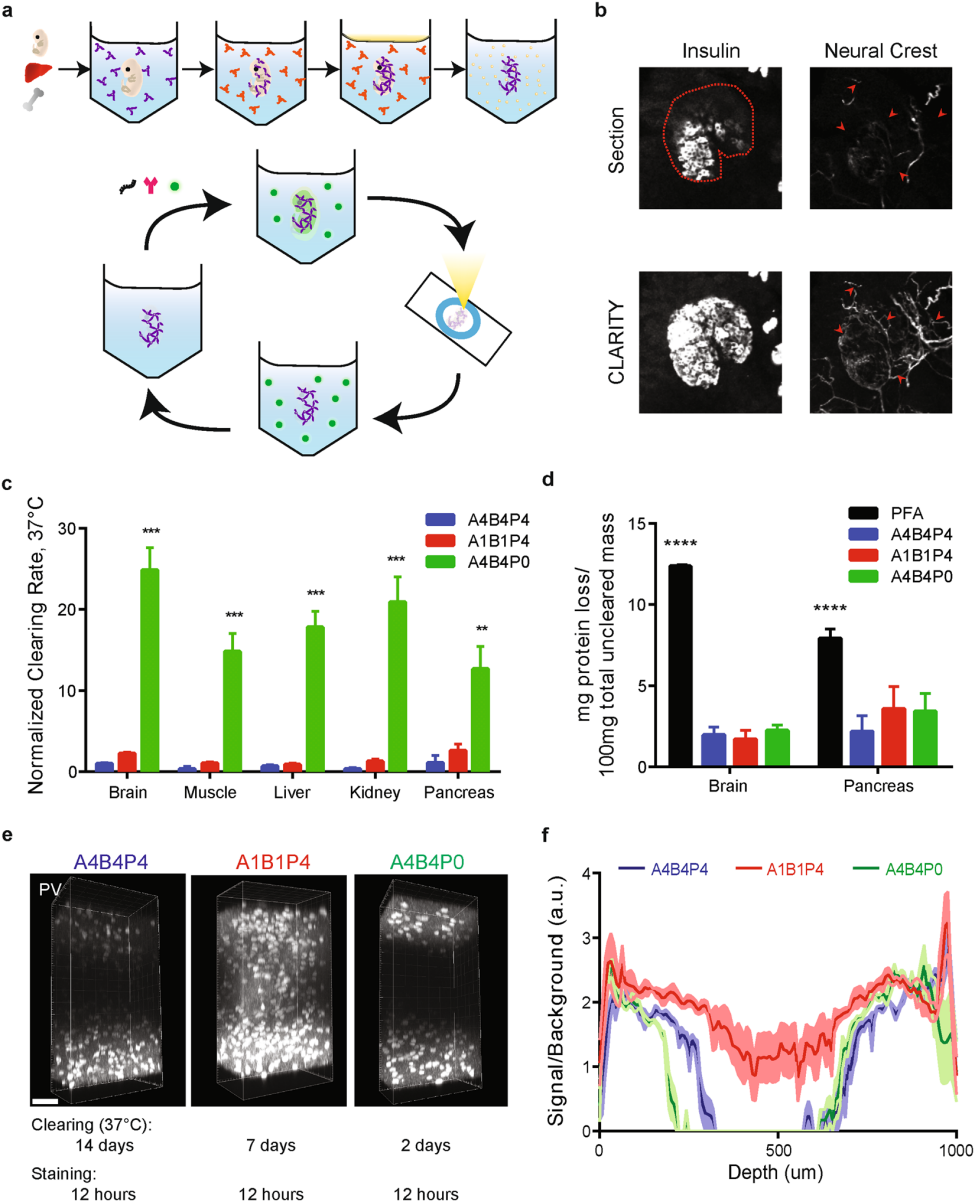
Development of enhanced CLARITY methods for peripheral tissues. (**a**) Simplified CLARITY schematic: First, fixed tissue is placed in a hydrogel monomer solution (purple) that diffuses throughout the tissue. Second, prior to polymerization, samples are transferred to a second hydrogel solution without the capacity for solid polymer formation (orange). Third, the sample is polymerized either through nitrogen flush and degassing or with the use of oil on the surface (indicated in yellow) as an oxygen inhibitor, resulting in formation of a tissue-polymer hybrid with a solid hydrogel interior but in liquid solution, which is ideal for fragile or irregularly shaped tissues. Fourth, tissue is passively cleared. Finally, after optical transparency is achieved, tissue is labeled with molecular probes (RNA, antibody, or dye), mounted on a slide in a refractive index matching solution, and imaged using standard microscopy. The sample can be destained and the process repeated for multiple staining and imaging cycles. (**b**) Virtual thin section (5 μm) compared to CLARITY volume (250 μm maximum intensity projection) of the same pancreatic islet image, here from a representative islet in a whole P15 pancreas from a Wnt1-Cre; ROSA-mTmG mouse. CLARITY enables more comprehensive and accurate measurements of structural features compared to section histology. Thin sections can grossly underestimate pancreatic islet sizes (dotted line), and results in incomplete observation of fine microstructures such as complex neural projections (arrowheads). (**c**) CLARITY gel A4B4P0 (green) clears significantly more rapidly compared to both A1B1P4 (red), and A4B4P4 (blue) across multiple tissue types including brain, muscle, liver, kidney (p < 0.00005, unpaired t-test), and pancreas (p < 0.0005, unpaired t-test). n = 6 sections per tissue type and gel. Clearing reaction rates were determined by fitting UV-spectrophotometry measurements over time to a first order transformation reaction kinetics equation y = Y_max_(1 − e^−kt^) and normalized to Brain A4B4P4. (**d**) Different CLARITY gel formulations do not impact the loss of protein in a cleared specimen compared to PFA-alone fixed tissue, as determined by BCA assay (p < 0.00005, unpaired t-test). n = 6 samples per tissue type and gel. (**e**) The A1B1P4 formulation improves the penetration rate of antibodies, resulting in full 1 mm brain slices stained with 12hrs incubation in antibody, visualized using the marker Parvalbumin (PV). Scale bar = 100 μm. (**f**) Staining quality from (**e**) was quantified by measuring average cellular signal intensity normalized by average extracellular background intensity. n = 6 samples per gel type. For all charts, error is s.e.m. **p < 0.005, ***p < 0.0005, ****p < 0.00005.

CLARITY and other hydrogel-based clearing techniques have successfully enabled 3D volumetric imaging at high resolution in mouse tissues such as brain, spleen, and bone^8, 19^. However, we found that when published CLARITY methods – which had been developed for solid organs with fixed architecture, such as the brain – were applied to irregular or fragile tissues such as clinical biopsies, significant difficulties were encountered. Most importantly, the firmness of the hydrogel composite (advantageous for many applications) created difficulties with friable tissues, resulting in clumping, shearing, and poorly-controlled gelation following hydrogel embedding. In turn, these effects led to adverse consequences including optical aberrations, reduced antibody penetration, high levels of tissue-gel composite expansion, and significant tissue damage, particularly in organs with irregular surface features or cavities where expansion could lead to rupture of enclosing structures. To overcome these challenges, we sought to re-engineer CLARITY for this new application domain, beginning by formulating a double matter phase (biphasic) hydrogel consisting of a solid gel within the tissue parenchyma but a liquid phase elsewhere including at irregular tissue and cavity boundaries, thus achieving mechanical stability of soft and friable tissue samples including clinical biopsies and mouse embryos (Fig. 1a). Subsequent volumetric imaging then enabled accurate visualization of fine anatomical structures that would otherwise be under-sampled or missed by thin section histology, including pancreatic islet volumes and neuronal structures (Fig. 1b).

To optimize biphasic CLARITY, individual components of hydrogel chemistry were titrated systematically with the goals of increasing clearing speed and retaining sample structure in soft tissue (Fig. 1c–f; Supp. Fig. 1). Acrylamide polymer (A), bis-acrylamide crosslinker (B), and paraformaldehyde fixative (P) are typically used in a fixed-ratio formulation abbreviated here as A4B4P4 (Methods); consistent with prior reports^19, 20^, we found that eliminating fixative while maintaining polymer and crosslinker (A4B4P0) could accelerate tissue clearing in many organs by 15–30x without affecting protein loss (Fig. 1c,d, Supp. Fig. 2), and up to 50x when cleared at elevated temperatures (Supp. Fig. 1). However, A4B4P0 gels were found to be prone to antigen loss when cleared for prolonged time periods (Supp. Fig. 2), which could limit utility for large tissues or for multiple rounds of immunostaining wherein tissues must be incubated in stringent elution conditions to remove antibodies^8, 9, 16^.

Consistent with previous reports^21^, reducing polymer and crosslinker concentration without altering fixative (A1B1P4) was found to modestly increase clearing kinetics 2–3x (Fig. 1c), and clearing rate could be further increased (15–30x) at higher temperatures (Supp. Fig. 1). These A1B1P4 gels result in clear 2 mm-thick tissue in 2–5 days without evidence of undue protein loss from excessive clearing (Supp. Fig. 2), and furthermore exhibited improved penetration of macromolecular probes; 1 mm-thick blocks of A1B1P4 composites could be stained homogeneously with high signal-to-background after only 12 hours incubation in antibody solution (Fig. 1e,f) compared to 48 hours previously reported for other CLARITY gel formulations^8, 20^. Thus for biphasic CLARITY, A4B4P0 composites are suitable for rapid clearing and a single round of antibody staining, but A1B1P4 composites may be suited for rapid single-round staining or multiple rounds of staining due to improved protein retention and greater rates of antibody penetration.

### Application to dynamics of neural crest cell progeny in the developing mouse pancreas

We next sought to utilize the novel biphasic CLARITY gel to investigate whole-organ pancreatic islet development at high resolution. During normal prenatal to postnatal gut development, neural crest derivatives and pancreatic islets develop in parallel^22–27^. Adult islets are innervated by sympathetic, parasympathetic, and nociceptive neurons, and are encapsulated by a sheath of peri-islet Schwann cells, all derived from neural crest progenitors^28^. Several lines of evidence suggest existence of postnatal remodeling of pancreatic islets through waves of beta-cell expansion and apoptosis^29, 30^, but the development of islet-associated neural crest cells has been investigated primarily in early embryogenesis^25^. The dynamics of these structures through the course of late-embryonic and postnatal development, when intact tissue volumes become too large for traditional imaging techniques, remain unclear. Recent studies of neurovascular development in pancreatic islets^27^ have proposed a model of monotonically increasing islet innervation in which autonomic nerves that initially line the surface of developing islets steadily penetrate the islet core throughout the first 2 weeks of life. Additional work has inferred relationships between islets and surrounding structures from high-resolution thin-section analyses^31–33^, optical tomography^34^, or 3D confocal reconstructions of individual islets^7, 35^, but studies with both high spatial resolution and whole-organ coverage have remained impractical with existing techniques.

**Figure 2:**
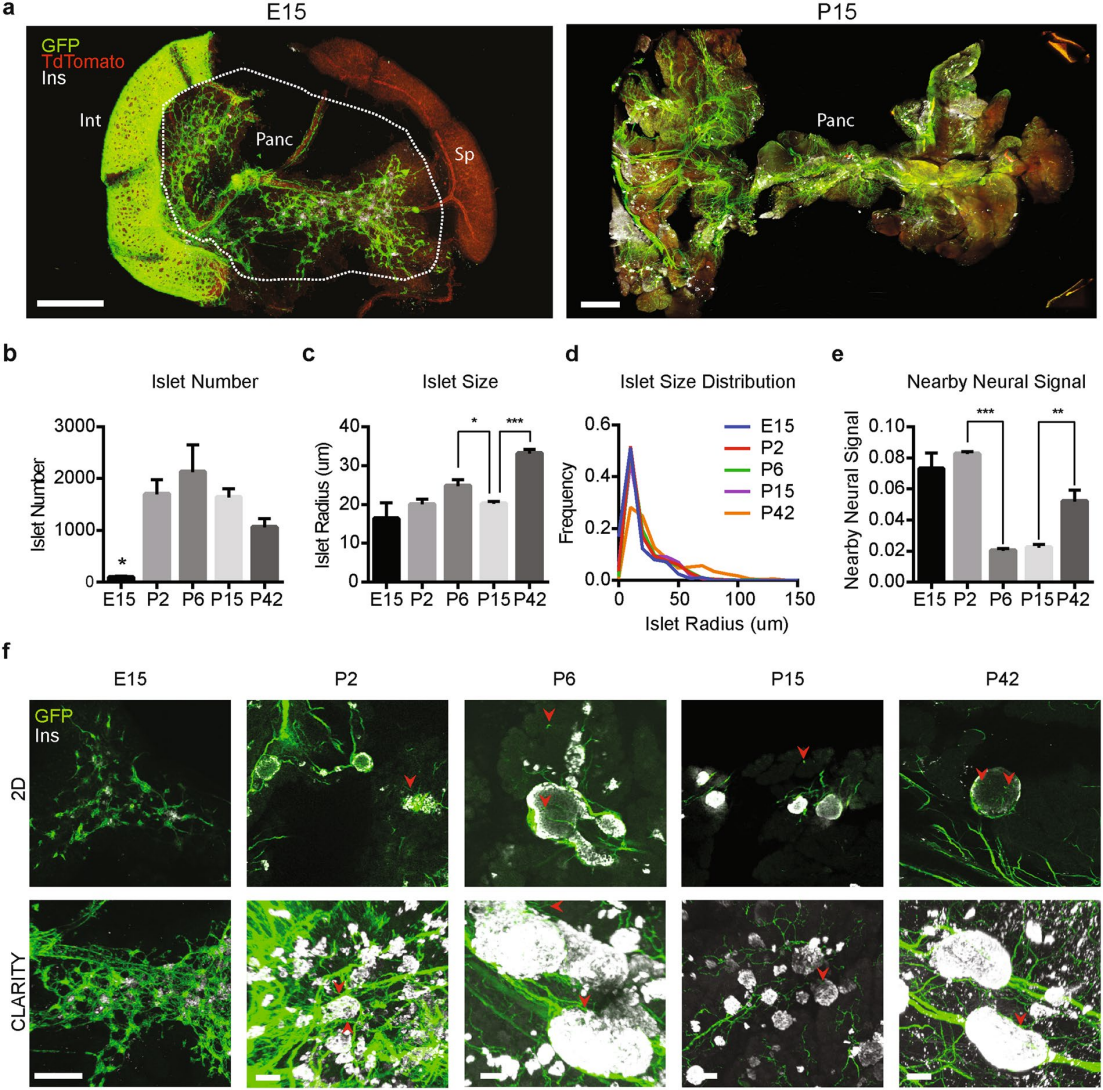
Neuroendocrine pancreas in mouse development. (**a**) Pancreata from Wnt1-Cre x Rosa26-mT/mG reporter lines at E15 (left) and P15 (right) were harvested, processed using CLARITY, and stained for insulin. 3D histology enables organ-wide visualization of developing islets (white) and associated neural features (green) throughout the pancreas; imaging datasets were then automatically processed with the computational pipeline (see Fig. S2). Int: intestine, Panc: pancreas, Sp: spleen. All samples were embedded with A1B1P4 hydrogel and cleared for 1–2 weeks at 37 °C. Scale bars = 1000 μm. (**b**) Automated quantification reveals changes in islet size and number at various stages of pancreatic development. Note massive expansion of islet number shortly following birth (p < 0.05, unpaired t-test) through P6, followed by reduction. (**c**) Islet size is also non-monotonic with aggregate size reduction (P6–P15, p < 0.05, unpaired t-test) and growth (P15–42, p < 0.0005, unpaired, t-test). (**d**) CLARITY enables refined whole-population measurements, as illustrated by a population shift towards larger islets at P42 compared to other timepoints. (**e**) The presence of neural crest structures in the neighborhood surrounding and within pancreatic islets was quantified. The level of neural crest-islet interaction is dynamic with a significant decrease between P2 and P6 (p < 0.0005, unpaired t-test) and a significant increase between P15 and P42 (p < 0.005, unpaired t-test). (**b**–**e**) n = 3 pancreata from litter mates per developmental stage, error is s.e.m. (**f**) Optical 2D sections (5 μm thick; top row) and corresponding 3D CLARITY images (1000 μm thick, bottom row) across development. While 2D sections suffice to observe gross changes in islet size, neural structures (arrowheads) are sparse and difficult to quantify in 2D. CLARITY reveals high-resolution structure not captured by 2D images, enabling visualization of neural remodeling dynamics. Scale bars = 100 μm.

To investigate and quantify interactions between islets and neural crest derivatives during development, pancreata were harvested at multiple fetal and post-natal stages from an established mouse strain used for labeling neural crest progeny (Wnt1-Cre; Rosa26-mT/mG)^18^, processed with biphasic A1B1P4 CLARITY, and imaged with confocal microscopy (Fig. 2a). This mouse line expresses a ubiquitous reporter in the form of a membrane-bound tdTomato that is converted to a membrane-bound GFP in the presence of Cre-recombinase, here driven by Wnt1, a neural crest lineage marker^36^—thus labeling cell membranes with GFP if of Wnt1 (neural crest) origin, and with tdTomato otherwise. Processing code (Supp. Fig. 3; Methods) was developed to identify insulin^+^ islet β-cells and neural crest derivatives, and to quantify colocalization. This system enabled rapid and unbiased population-level characterization of islet association with neural crest derivatives (Fig. 2b–e). At each developmental stage, islet radius and the extent of neural crest signal in and around the islet were quantified for each detected islet. The ability of the software to accurately detect population-wide changes in both islet size and neural-crest derivative signal was validated using a known model of drug-induced diabetes, which results in beta-cell death and reactive gliosis (Supp. Fig. 4)^7, 37^.

Consistent with previous reports^38, 39^, marked increases in both islet number and size between mid-gestation (E15) and the neonatal period (P2 to P6) were observed, followed by a modest reduction in total beta-cell volume at P15 before continued growth (Fig. 2b,c), potentially due to apoptosis, formation of new islets, or other mechanisms. From automated measures of all pancreatic islets, population distributions of islet sizes during development could also be constructed. As expected, the proportion of islets with larger sizes rose throughout postnatal development (Fig. 2d). Intriguingly, quantification of islet-associated nearby neural signals revealed evidence of changing neural-islet interactions. A reduction in peri-islet neural crest signal between P2 and P6 was followed by a gradual rebound in levels of innervation and glial encapsulation by P42 (Fig. 2e,f). These data revealed unexpected multiple dynamics of islet interactions with neural crest-derived structures, potentially due to pruning, the birth of new under-innervated islets, or other phenomena.

### Biphasic CLARITY for quantification of structures spanning whole human organs

We next sought to determine if the developmental dynamics observed in mouse would be conserved in human development. Such a cross-species correspondence would be far from certain *a priori*, particularly since pancreatic innervation during development in humans, while closely associated with islets^40^, has been reported to deviate significantly from that in rodents^32, 33^.

**Figure 3:**
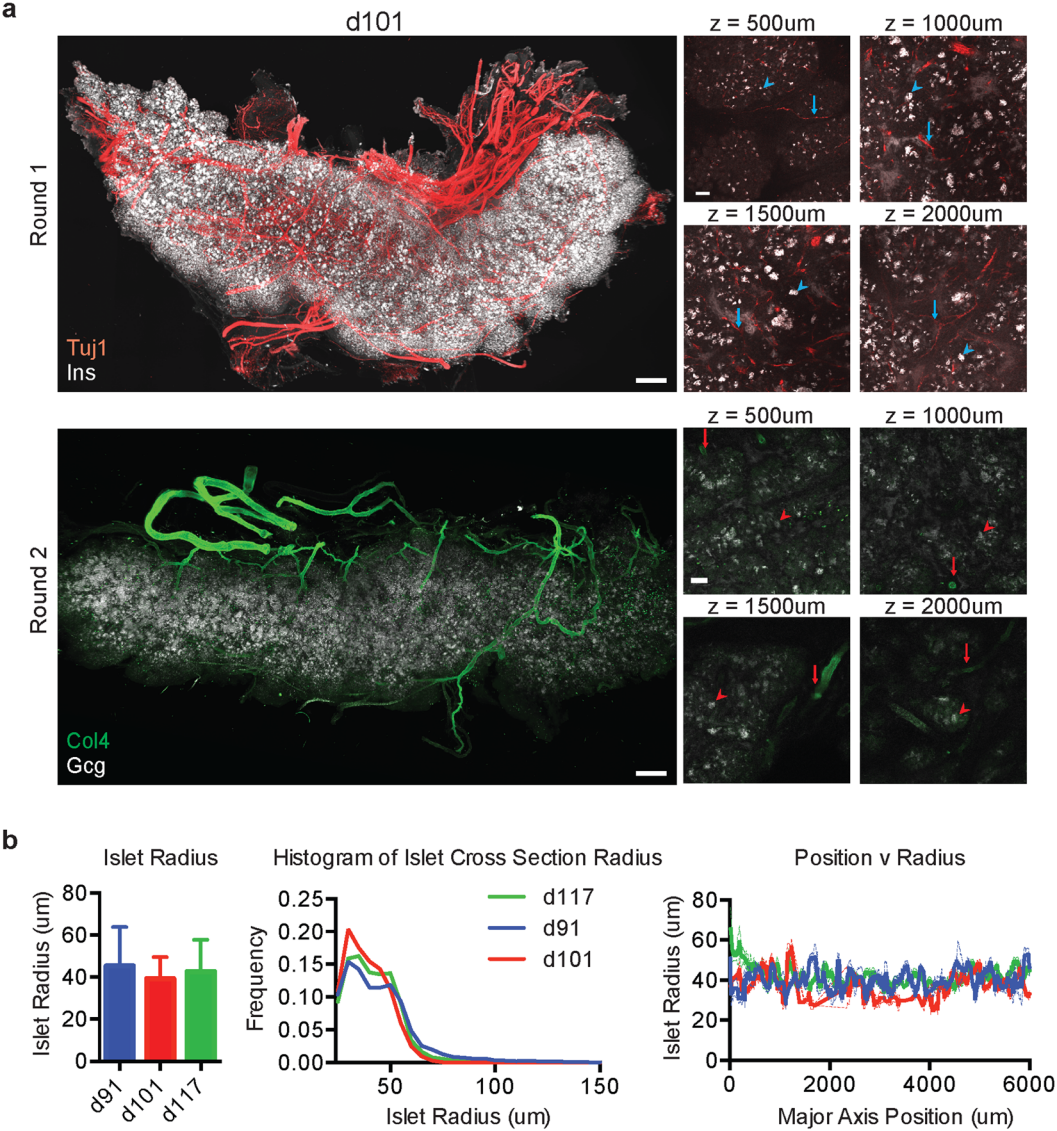
CLARITY for whole human organs. (**a**) CLARITY enables high-resolution and high-quality whole-organ tissue processing and analysis of human fetal tissues. A day 101 fetal sample (top row) can be stained, with Insulin (Ins) and β-tubulin (Tuj1), and imaged through several millimeters of cleared tissue without loss of resolution. Four example z positions spanning the entire pancreas thickness are shown with islets (arrowheads) and nerves/vessels (arrows) observed at each depth. The same tissue can be stained multiple times with different sets of antibodies – Type 4 Collagen (Col4) and Glucagon (Gcg) - (bottom row) with minimal loss of feature resolution. Scale bars = 500 μm whole sample, 100 μm inset. Samples were embedded in A1B1P4 hydrogel and cleared for 2–3 weeks at 60 °C. Antibody removal was performed by clearing for 5–7 days at 60 °C. (**b**) CLARITY enables automated quantification of structural features, including islet size. In this cohort, there was no significant difference (unpaired t-test) in average islet size, population distributions, or spatial distributions of islet sizes at different developmental ages between d91 and d117. Error: s.e.m. n = 3.

To assess islet innervation during human development, we embedded human fetal pancreatic tissue (91–117 days post-conception) in biphasic A1B1P4 hydrogel and clarified the samples at 60 °C for 2–3 weeks. With standard confocal microscopy (Methods), this approach enabled high-resolution imaging of fine immunolabeled structures, including islet cells and neurons as labeled by antibodies against Insulin (Ins) and β-tubulin (Tuj1), throughout millimeters of clarified tissue (Fig. 3a). Furthermore, multiple rounds of immunostaining were feasible with this approach in large blocks of human tissue and whole human organs, enabling visualization of different islet cells and vasculature as labeled with antibodies against glucagon (Gcg) and Type-4 collagen (Col4) (Fig. 3a). Initial automated quantification of thousands of islets in each sample revealed no significant sample variation or change in mean islet size within the d91-d117 range (Fig. 3b). Although asymmetries might be anticipated between the head and tail of the pancreas, which have distinct developmental origins^41^, spatial heterogeneity was not detected at this stage of development across all 6 mm of tissue from head to tail (Fig. 3b).

**Figure 4:**
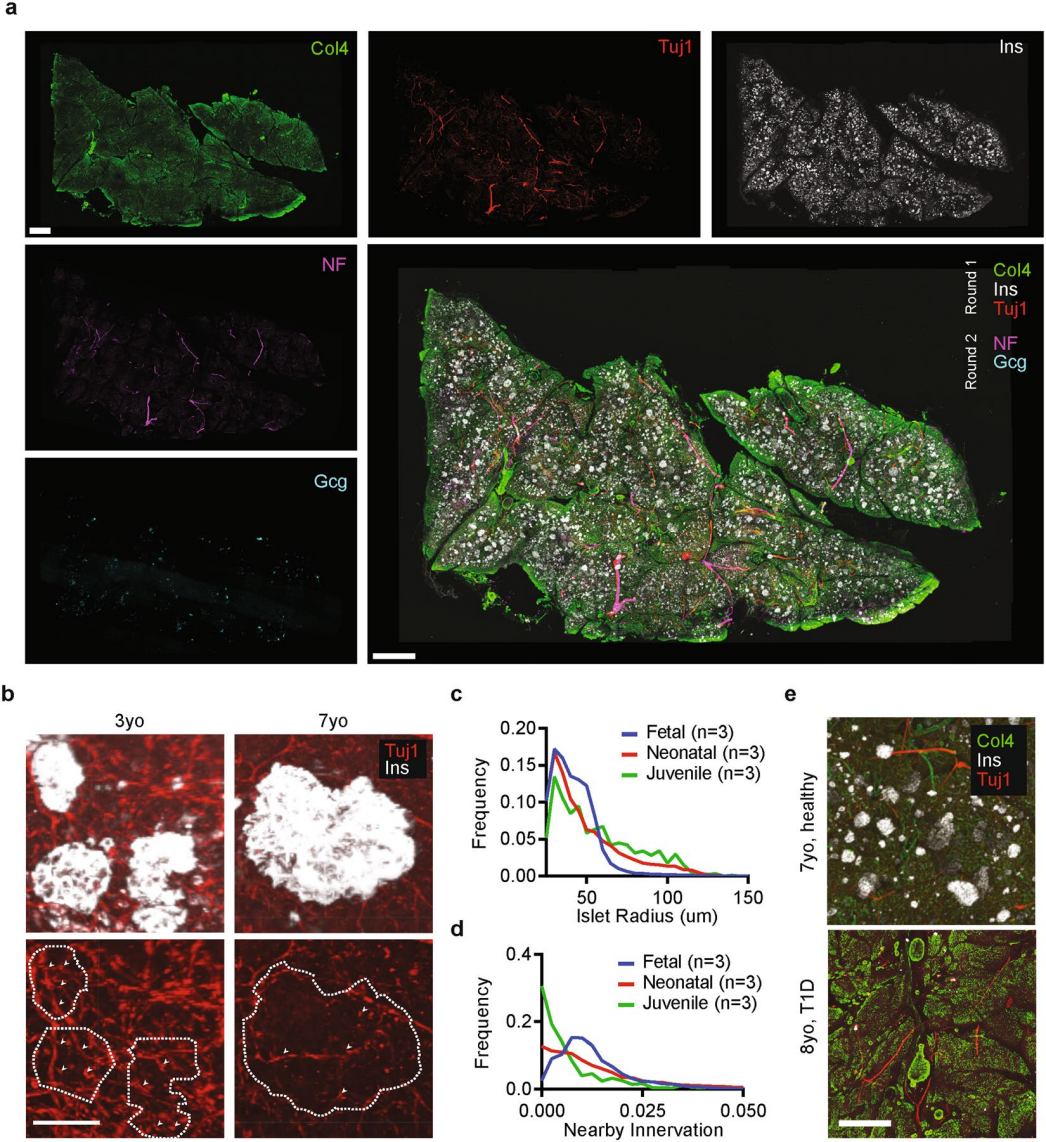
Neuroendocrine pancreas in human development. (**a**) CLARITY enables multi-round immunolabeling of large human tissues, such as in this 1 mm thick sample from a 3 year old human pancreas stained for Type 4 Collagen (Col4), β-tubulin (Tuj1), Insulin (Ins), Neurofilament (NF), and Glucagon (Gcg) over two rounds and manually registered together using image features. Samples were embedded in A1B1P4 hydrogel and cleared for 2–3 weeks at 60 °C. Scale bar = 1000 μm. (**b**) 3D histological examination of two human pancreas samples at age 3 years and at 7 years illustrate differences in islet size (dashed lines) and innervation (arrows) during postnatal human development. Scale bar = 100 μm. (**c**) Analysis of islet populations across fetal (n = 3), neonatal (n = 3, 0–3yo), and juvenile (n = 3, 4–8yo) human samples reveals a distribution shift toward larger islets as humans age. (**d**) Analysis of innervation surrounding and within islet populations across fetal, neonatal, and juvenile human samples reveal distribution shift toward reduced innervation in islet neighborhoods during postnatal human development. (**e**) CLARITY may enable assessment of relevant features in diseased tissue. Here, an 8yo subject with history of T1D shows minimal evidence of insulin-expressing islets compared to healthy age-matched control. Samples were cleared and stained under identical conditions but in separate experiments. Scale bar = 500 μm.

To quantify postnatal innervation patterns of pancreatic islets in human samples, we performed biphasic CLARITY on pancreatic tissue from pediatric donors (2–8 years of age) and immunolabeled with antibodies against Ins, Gcg, Tuj1, Col4, and Neurofilament (NF) (Fig. 4). Consistent with our mouse tissue results, clarifying at elevated temperatures was rapid and resulted in structurally sound tissue blocks spanning many millimeters in cross-section and up to 2 mm thick. These tissue blocks could be probed sequentially with multiple rounds of antibody labelling, with image stacks subsequently registered together to create high-dimensional phenotypic datasets (Fig. 4a). Crucially for clinical relevance, we found the CLARITY process was compatible with recently-prepared PFA-fixed (Fig. 3) and flash frozen specimens (Supp. Fig. 5) as well as with archival blocks of fixed pancreatic tissue (Fig. 4). Automated analysis of both prenatal and postnatal human pancreata revealed that as subject age increased from fetal to neonatal to juvenile, beta-cell clusters became larger (consistent with prior human studies^40^) and also less invested with neural elements (Fig. 4b–d) on a population-wide basis, consistent with the postnatal neural remodeling observed in mouse models.

These tissues had no known intrinsic disease process. To test utility of biphasic CLARITY for pathological human specimens, with potentially different tissue properties due to chronic inflammation, fibrosis, or other disease-related responses^42^, identical processing was performed on samples obtained from pediatric subjects with multi-year histories of type 1 diabetes mellitus, revealing catastrophic loss of insulin-expressing cells and islets pathognomonic for type 1 diabetes mellitus (Fig. 4e)^43^. Additionally, we confirmed that biphasic CLARITY was broadly applicable to numerous banked tumor biopsies representing a wide range of cancers, and enabled visualization and demarcation of complex three-dimensional tumor boundaries (Supp. Fig. 6). Finally, we tested whether CLARITY-processed tissue could be subsequently used for traditional histopathology inquiries, and found that indeed clarified human tissues could be sectioned and stained with hematoxylin/eosin to reveal structures typical of current histopathological evaluation (Supp. Fig. 7).

### Quantitatively improved accuracy of histology via volumetric biphasic CLARITY

Whole pancreata could be analyzed with biphasic CLARITY to assess islet and neural crest interactions at a population-wide level, providing measures of phenotypic distribution that previously had only been modeled theoretically^44^. Beyond opening a path to new histopathological datastreams, we wanted to directly test if an intact volumetric approach could furthermore provide more accurate measurements versus two-dimensional histology. Measuring the number and the 2D width of objects in thin sections is crucial for a vast array of staging and diagnostic purposes^45, 46^, but is known to suffer from systematic bias depending on the sectioning plane and individual sample features that may happen to be available on the selected slide^47–51^.

**Figure 5:**
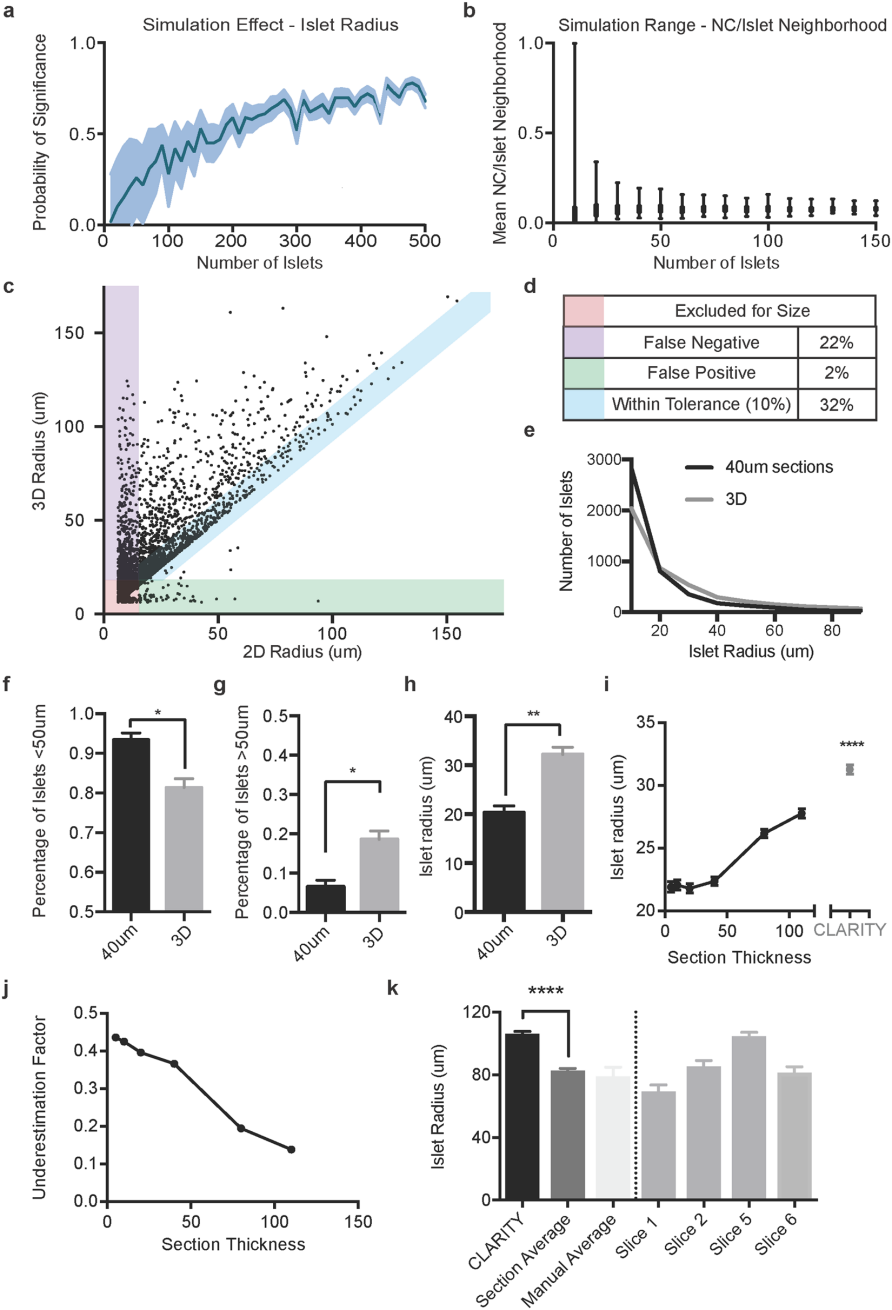
Computational demonstration of quantitative advantage of organ-wide 3D histology. (**a**) CLARITY enables automated analysis of large populations that provide greater statistical confidence. In a simulated experiment measuring islet radius, N islets were randomly selected, with replacement, from 3 P6 pancreata and 3 P15 pancreata CLARITY datasets, and islet radius means were compared. The proportion of experimental comparisons that reached statistical significance (p < 0.05, unpaired t-test) when the experiment was performed 100 times is plotted against the number of islets measured per experiment. When sampling the size of approximately 250 islets in each pancreas, there is only a 50% chance the data will be significant even though the true trend exists. (**b**) Large populations greatly reduce experiment-to-experiment variability. Bars show the range of the mean of N islets when a simulation measuring islet size and neural crest interaction was repeated 100 times. The width of the experimental ranges, and hence experiment-to-experiment variability, is reduced with greater sample size per experiment. (**c**,**d**) CLARITY provided more accurate measures of islet size compared to 2D analysis. A whole pancreas was analyzed using CLARITY or eight non-adjacent 40 μm optical sections, and raw values for islet radius plotted. Features less than 10 μm were excluded from analysis due to being smaller than an islet (red). False negative values, where 2D estimation of radius would have incorrectly excluded the islet when the 3D size was large enough for analysis, represented 22% of the remaining islets (purple). False positive values, where the islet would have been excluded in 3D but were measured in the 2D analysis, represented 2% of the islets (green), and only 32% of the islets had sizes estimated in 2D that were within 10% of their 3D size. The remainder (68%) of measurements were underestimations of greater than 10%. (**e**–**g**) CLARITY provides more accurate measurements of islet number. A 2D and 3D analysis of the same sample demonstrated a significant excess of islets < 50 μm in sectioning, but undercounting of islets > 50 μm. (n = 3) (**h**) CLARITY can eliminate measurement errors due to sectioning. For each islet, 2D and 3D size was calculated. 2D sectioning significantly underestimated average islet size due to sectioning away from the islet equatorial maximum compared to volumetric analysis. (**i**) To assess the effect of 2D section thickness on islet radius measures, simulated optical sections of a range of slice thicknesses were analyzed. All 2D sections significantly underestimated islet radius compared to the measure in 3D. (**j**) Underestimation of islet size in 2D was calculated compared to 3D by taking the difference between values and dividing by size in 3D. 2D slice-based methodologies underestimate islet size by nearly 40%. (**k**) To explore whether error could be overcome by examining only large structures, the largest 20 islets in each CLARITY and 2D section (or total islets, if <20 were found) were averaged and compared for size. In addition, 20 islets were manually independently and blindly selected and measured in each section. 2D histology systematically underestimated islet radii in even the largest islets by >20% (p < 0.0001, Mann-Whitney test). 4 individual 2D sections shown to illustrate size variability by slice.

A significant advance enabled by volumetric histology could potentially be the ability to perform unbiased, organ-wide analysis of arbitrarily large numbers of structures, rather than reliance on manual selection of a small subset of features, thereby significantly enhancing statistical power and reducing sample variance. To quantify this phenomenon in our data, we performed multiple simulated experiments of islet measurement on the same dataset–comparing islets from different developmental stages, while increasing the sample size from tens (as would be the case if performed manually) to hundreds or thousands (straightforward with the automated CLARITY workflow but otherwise prohibitively labor intensive). As hypothesized, we found that random selections of islets failed to uncover significant size differences between populations unless hundreds of islets were sampled (Fig. 5a). Further, we found that experiments measuring 30 islets or fewer in a given sample (in line with most published studies in this field) resulted in significant experiment-to-experiment variation in population measurements of neural signal, but as expected, as the number of measurements enabled by population-level analysis increased to hundreds, experimental variation was drastically reduced (Fig. 5b).

Another (potentially even more significant) advantage enabled by volumetric histology could be an ability to observe the entirety of an anatomical structure rather than a 2D cross-section. To explore this advantage, we directly assessed measurement bias in comparison with the automated volumetric approach by extracting virtual 2D histology sections directly from 3D human juvenile pancreas images, thereby crucially comparing the very same samples against themselves using both approaches. We compared islet radius in 40 μm optical sections to the same islet measured in 3D (Fig. 5c). In our analysis, we set a threshold of 10 μm – approximately the diameter of one endocrine cell^52, 53^ – as a typical minimum radius to qualify an object as an islet, as anything smaller in this preparation could be excluded (representing staining artifact or non-cellular feature).

Of the 4564 islets analyzed in both 2D and 3D, we found that 22% would have been incorrectly excluded due to insufficient size in 2D sections, even though the islet size as measured in 3D was above threshold (reported as “false negative”, Fig. 5d). Strikingly, only 32% of islets measured in 2D were within 10% of the ground-truth 3D size value (reported as “within tolerance”, Fig. 5d). Thus the vast majority of 2D islet measurements dramatically underestimate islet radius. Furthermore, the number of small islets is overestimated and large islets are underestimated in 2D sections, as a result of sectioning away from the islet equator leading to incorrect radius measurements (Fig. 5e–g). The combined effect of these multiple sources of error is to substantially underestimate average islet radius when measuring in 2D compared to 3D (Fig. 5h), which would have even more-radical impact on the most clinically relevant estimates of beta-cell mass and volume (as the error is cubed).

We next explored if changing the thickness of 2D sections could reduce the inherent errors in islet number and size, and generated optical sections for a variety of slice thicknesses quantifying 2D islet radius compared to volumetric analysis (Fig. 5i). All 2D slices underestimated islet radius, with decreasing error as the slice thickness approached the size of the 3D volume. For each islet measured in 2D, we calculated the percentage error compared to the 3D value (Fig. 5j). A 5 μm section – the thickness most commonly used in clinical evaluation^54^ - underestimated islet size by more than 40%, and 40 μm sections systematically underestimated islet size by more than 35%.

We considered the possibility that the majority of this error was driven by small islets, where slight measurement discrepancies could have an outsized impact on percentage-based error. However, even when selecting only the largest islet cross-sections, as might be examined in experimental and clinical settings, section-based analysis underestimated the average islet radius by up to 20–30% (Fig. 5k). Hence, even for approximately spherical islets, among the simplest geometric features found in anatomy, 2D analysis is insufficient to provide accurate measurements of means or distributions of sizes for fundamental structural features.

Taken together, these simulations offering the first direct quantitative comparisons between 2D and 3D histology data demonstrate substantial improvements in experimental variation, statistical power, and measurement accuracy when using three dimensional analyses like CLARITY. Further, while these analyses were performed for islets distributed within exocrine pancreas, the results would be extensible to detection of tumor microsatellites within healthy tissue, measurement of tumor dimensions and boundaries, and comparison of morphological criteria important for tumor staging/typing across populations of neoplastic cells.

### Biphasic CLARITY: broadly applicable to multi-organ and whole-organism studies

**Figure 6:**
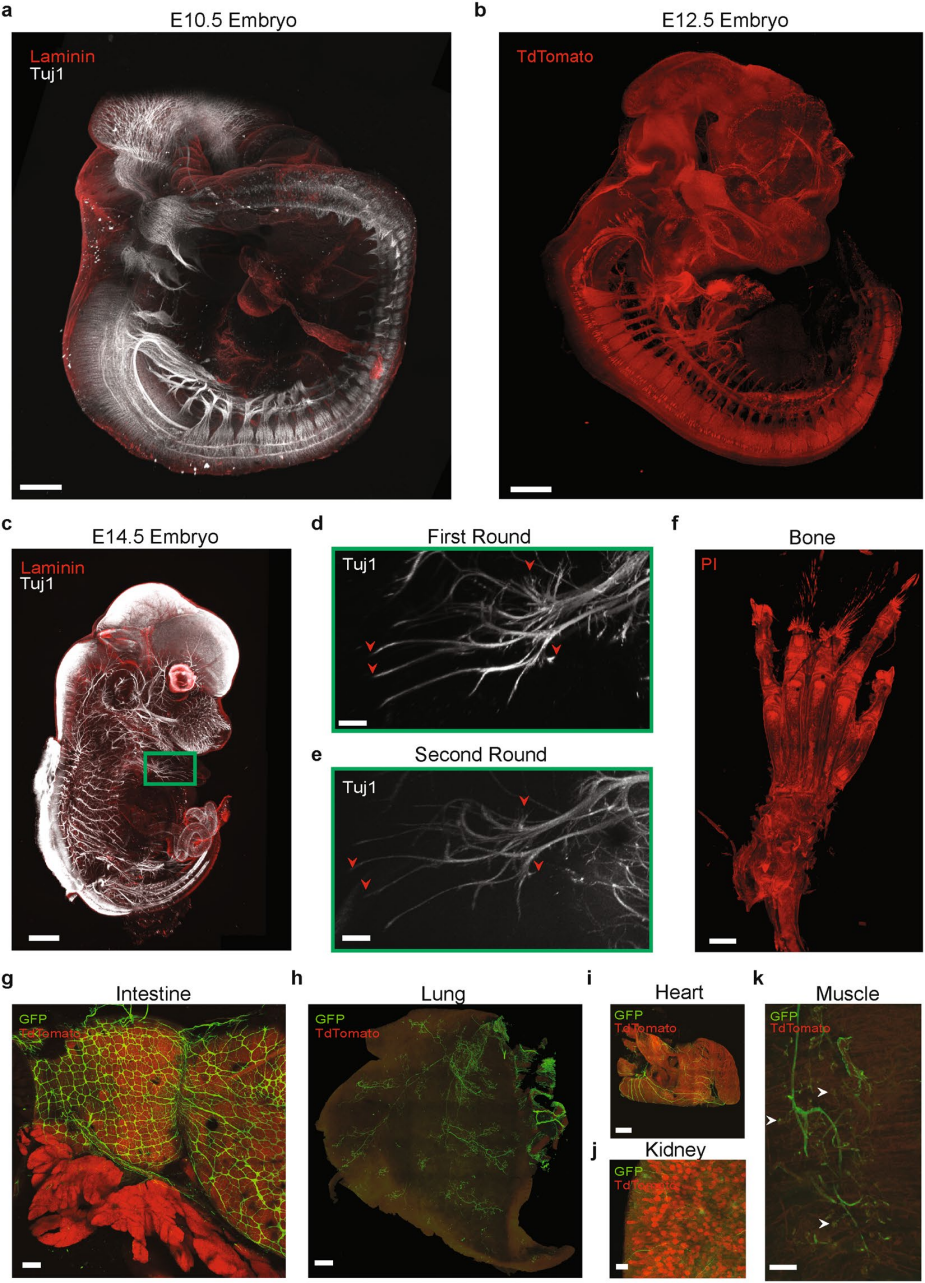
Applications of CLARITY to diverse biological systems. (**a**) Wildtype E10.5 mouse embryo was cleared and labeled with Tuj1 and laminin. Scale: 500 μm. (**b**) E12.5 Embryo from Wnt1-Cre x Rosa26/TdTomato mouse was clarified and imaged, demonstrating robust preservation of endogenous labels. Scale: 100 μm﻿. (**c**) Whole E14.5 mouse embryo was stained for Tuj1 and Laminin, the antibody label removed, and the specimen restained with the same antibody. Scale: 1000 μm. (**d**,**e**) Even after two rounds of staining, the same fine structural features (arrowheads) can be easily identified in the whole sample (green box). Scale: 200 μm. (**f**) CLARITY can successfully clear decalcified bone (see Methods). Scale: 1000 μm. Samples in panels a-f were embedded in A1B1P4 hydrogel and cleared for 2–4 weeks at 37 °C (see Methods and detailed protocol for tissue-dependent timing). (**g**–**k**) Other whole organs can also be cleared, including the gastrointestinal tract (here, pyloric sphincter, scale: 500 μm), lung (scale: 500 μm), heart (scale: 1000 μm), kidney (scale: 100μm), and skeletal muscle (scale: 50 μm). CLARITY preserves fine structures, enabling visualization of, for example, neuromuscular junctions (arrowheads). Samples in panels (**g**–**k**) were from adult Wnt1-Cre; Rosa26-mT/mG reporter lines, embedded in A4B4P0 hydrogel and clarified for 1–2 wk at 37 °C (see detailed protocol for tissue-specific timing).

The biphasic CLARITY and imaging techniques reported here were also found to be well-suited for whole-system studies typically too large for traditional imaging techniques, including late-stage mouse embryonic development (Fig. 6a–e) and adult organs (Fig. 6f–k). These protocols may therefore be applicable to numerous biological systems of interest to surgical pathology, developmental biology, and other areas of biomedicine requiring high-resolution intact maps of organ-wide architecture with automated quantification. Indeed, in adult tissues as diverse as bone, intestine, lung, heart, kidney, and muscle, this approach allowed observation of fine structures, such as peripheral and enteric nerve endings, with sufficient resolution to identify (for example) neuromuscular junctions (Fig. 6k; Methods). Finally, similar to the original CLARITY formulation, these biphasic hydrogels are compatible with multi-color immunolabeling, single- and multi-color endogenous fluorescence labeling, and multiple-round immunostaining with preservation of fine structures (Fig. 6d,e), all essential techniques for developmental biology and clinical diagnostics.

## Discussion

Histopathology is a fundamental diagnostic, prognostic, and treatment-guiding tool of modern clinical medicine. Accurate measurements of known biological features are a critical component of clinical work including cancer staging, and it is known that microscopic spatial differences can affect diagnosis and consequently treatment^46^. For example, evaluation of sentinel lymph node biopsies is critical for detection of microsatellites and micrometastases, yet guidelines recommend only sampling a single section per lymph node, or a maximum of one section per 2 mm, for evidence of malignancy^55^, resulting in published false negative rates of up to 5–10% or more^56^. Moreover, many diseased tissues are known to exhibit highly heterogeneous properties^48^–for example non-uniform mitotic activity across a tumor–but sampling bias from sparse observations can result in reliability as low as 40%^47^, a problem only compounded with more nuanced metrics such as gene expression or morphology.

Traditional methods requiring sectioning of samples into two-dimensional slices are inherently limited in ability to comprehensively and reliably characterize complex three-dimensional structures. Three-dimensional techniques, on the other hand, enable accurate and quantitative analysis of many sample types, including those with convoluted surface morphology not fully captured by section-based histology^45, 51, 57, 58^, such as tumor margins, low-grade abnormalities in glandular cell growth, angiogenesis patterns within tumors^59^, ductal integrity and morphology, and invasion depth of tumors into surrounding tissues.

Though other techniques exist for volumetric histology, including methods developed for human studies or clinical applications^60–62^, CLARITY has the advantages of retaining compatibility with nucleic acid and protein probes and small molecule dyes, enabling multiple rounds of staining within the same sample, preserving endogenous fluorescent signals, and permitting analysis of previously fixed and banked specimens. Critically, we provide the first evidence that CLARITY can be easily integrated into a standard clinical workflow by demonstrating compatibility with flash frozen and formalin-fixed human specimens, as might be obtained from typical autopsy or surgical pathology procedures, and post-CLARITY H&E processing.

However, additional opportunities remain for improvement in clarifying speed, antibody compatibility and penetration, and scalable analytic tools that can be generalized to diverse clinical applications. Indeed, much progress has been made along these lines for volumetric histology in research contexts, particularly for the mouse brain^9, 19, 63^–and emerging tools for direct clinical analysis from serial section efforts^64, 65^ could be adapted for whole-mount pathology. Additionally, the introduction of extremely large imaging datasets creates challenges for data management; indeed, though CLARITY is compatible with diffraction-limited imaging resolution (enough to, for example, delineate individual synapses and other subcellular structures^8, 16^), the whole-organ images here were acquired with cellular resolution, as needed for our analyses. Finally, though our experiences with CLARITY demonstrate the flexibility of the approach, there is no current blanket protocol that is universally optimized for all possible samples; we discuss guidelines for best practices (see Supplemental Material), but application-specific optimizations will still need to be tested by the end-user.

The advent of numerous tissue clearing techniques coupled with increased data processing ability reveals the potential for 3D digital pathology, enabling high-throughput, automated characterization of complex 3D tissue architecture and molecular properties in large volumes of clinical tissue, eliminating sample bias, and reducing the costs associated with labor and interpretation. High-resolution imaging datasets, combined with the growing capabilities of computer vision and machine learning, including in diagnostic contexts^66^, further set the stage for computer-guided analytics and discovery directly from clinical specimens.

## Methods

### Animals

All aspects of animal husbandry and experimentation were performed in accordance with guidelines from the National Institutes of Health and have been approved by members of the Stanford Institutional Animal Care and Use Committee. All animals were maintained on a standard light-dark cycle with access to chow and water *ad libitum*. Adult male and female C57BL/6 J mice were utilized for adult (6–8 weeks of age) and postnatal (less than 15 days of age) CLARITY experiments. Developmental stages were calculated as days from detected vaginal plug. Wnt1-Cre; ROSA-mTmG mice were generated by crossing the *129S4*.*Cg-Tg*(*Wnt1-cre*)*2Sor/J* mouse line (Jackson Labs, #022137) with the *Gt*(*ROSA*)*26Sortm4*(*ACTB-tdTomato*,*-EGFP*)*Luo/J* mouse line (Jackson Labs, #007576).

### Human specimens

Use of de-identified post-mortem human tissue was in accordance with guidelines from the National Institutes of Health and approved by the Stanford Institutional Review Board. Fetal pancreas samples were obtained from the University of Washington Birth Defects Research Laboratory, juvenile pancreas samples from the International Institute for the Advancement of Medicine, and frozen pancreas specimens from Cureline, Inc (South San Francisco, CA), with written informed consent from each subject obtained by each respective institution.

### Simplified CLARITY protocol (see supporting detailed protocol)

The A4B4P4 hydrogel formulation is as described previously^8, 16^, consisting of an aqueous solution of 4% Acrylamide (wt/vol), 0.05% Bis-Acrylamide (wt/vol), 4% Paraformaldehyde (wt/vol), and 0.25% VA-044 initiator (wt/vol) in PBS. A1B1P4 hydrogel formulation reduces the 4% acrylamide to 1% and the 0.05% bis-acrylamide to 0.0125%. A4B4P0 formulation eliminates PFA in the hydrogel solution, although PFA is still present during animal perfusion.

Animals were deeply anesthetized and perfused transcardially with phosphate buffered saline solution (PBS, Thermo-Fisher, pH 7.4), followed by perfusion of an equal volume of cold 4% PFA, and post-fixed in PFA overnight at 4 C. Samples were then incubated in various hydrogel solutions at 4 C for 2–4 days to allow sufficient hydrogel diffusion into the tissue. Human tissue samples were similarly fixed in PFA, then transferred to cold hydrogel for 2–4 days.

For applications necessitating liquid hydrogel formulations, the solution was replaced with a non-rigidly-polymerizing hydrogel solution (such as A1B1P4, A4B0P4, or PBS) immediately before embedding (Supplemental Tables 1–5). As a result, tissue is sufficiently crosslinked to maintain structural stability, but the surrounding solution remains in liquid phase.

To embed tissues into hydrogel, tissues were polymerized either following the nitrogen-flush and vacuum chamber degassing protocol previously detailed^8, 16^, or in the simplified protocol, by covering the tissue-containing solution with a layer of hydrophobic oil (we used castor oil [Sigma 259853]) without the need for degassing. All samples were polymerized by incubation in a 40 C shaking incubator for 3–5 hours, followed by removal of excess hydrogel. Solid gels can be peeled from the sample under a fume hood and disposed of as solid waste; liquid gels can be poured directly into liquid waste.

Samples were then placed in clearing solution of 4–8% sodium dodecyl sulfate (SDS) in either Sodium Borate buffer (200 mM, pH 8.5) or, in the simplified protocol, PBS (pH 7.4), in a shaking incubator set at 37–60 °C (Supplemental Tables 6–8). Samples were passively cleared at either 37 °C or 60 °C until optical transparency was achieved, as shown in Fig. 1c (Supplemental Table 9). Before further processing, clearing solution was rinsed from the sample with 2–3 washes of PBST (0.1% TritonX-100 (wt/vol) in PBS) over 1 d.

### Quantified clearing rate

Mouse organs were harvested as described above and coarsely sliced into 2 mm-thick blocks after polymerization. Samples were placed in 4% SDS clearing solution at the indicated temperature (Fig 1d, Fig. S2). Optical transparency was assessed using UV spectrophotometry (BioTek, Epoch 2 Microplate Spectrophotometer). Clearing rate was determined by fitting a one-phase exponential association equation of the form *y* = *Y*_*max*_ (1 − *e* ^*−kt*^), chosen because tissue clearing can be modeled as a single-reactant transformation reaction with first-order rate kinetics. Rates were normalized to A4B4P4 brain tissue cleared at 37 °C to quantitatively determine relative rates compared to previous CLARITY protocols.

### Protein loss

Samples were coarsely sliced into 2 mm blocks after polymerization and weighed prior to clearing. Tissues were allowed to clear in clearing solution; at the indicated time points, 500 ul of solution was removed from the sample to assess protein loss. The quantity of protein loss was characterized using a bicinchoninic acid (BCA) protein assay (Pierce Biotech) following manufacturer’s instructions. Results were normalized by samples’ starting weights.

### Antibody penetration

Mouse brain samples were collected as described and sliced into 1 mm sections prior to clearing. Cleared samples were incubated for 12hrs at room temperature with rabbit anti-parvalbumin antibody (Invitrogen, 1:100 dilution) in PBST, washed 3 times in PBST, and incubated for 12hrs at room temperature with anti-rabbit (Fab)2 fragments conjugated to AlexaFluor 647 (Jackson, 1:100 dilution) in PBST. Samples were imaged at 40x using identical laser and microscope settings, and results are shown in Fig. 1f. Staining quantification was performed using custom Python code. Each image slice was thresholded using an Otsu threshold from the Skimage Python package. Positively stained cells were detected by eroding the image by one pixel using the Python Scipy package, followed by morphological closing, analysis of region properties measured with Skimage, and exclusion of non-cellular features based on size and roundness. Average region intensity, representing the average intensity of positively stained cells, was calculated by averaging pixel values within all detected regions in a plane, and background levels were obtained by averaging pixel values outside the detected regions. Signal over background was calculated by dividing the average pixel intensity in each region by the average background intensity.

### Immunostaining of CLARITY-processed pancreas

Samples – both whole mount rodent pancreas and 1 mm-thick human specimens - were incubated for 2 days at room temperature with primary antibody (1:100 dilution) in PBST, washed 3 times with PBST, and incubated for 2 days at room temperature with AlexaFluor-conjugated secondary antibody (Jackson, 1:100 dilution) in PBST. For immunostaining of samples derived from frozen clinical specimens, an identical procedure was applied except preceded by overnight blocking in 3% Normal Donkey Serum + 0.3% Triton-X in PBS at room temperature.

List of labeling reagents used in this study:

- anti-Parvalbumin (rabbit, Abcam ab11427)
- anti-Insulin (guinea pig, Dako A0564)
- anti-Tuj1 (mouse, Millipore MAB1637)
- anti-Col4 (rabbit, Millipore MAB8201)
- anti-GFP 488 (rabbit, Thermo-Fisher A21311)
- anti-Glucagon (guinea pig, Takara M182)
- anti-CD31 (rabbit, Abcam ab28364)
- anti-Laminin (rabbit, Millipore AB2034)
- anti-Somatostatin (rabbit, Peninsula T-4103)
- anti-melanA (mouse, Dako A103)
- anti-CK7 (mouse, Dako OV-TL 12/30)
- anti-CK20 (mouse, Dako Ks20.8)
- Propidium Iodide (Cell Signaling Technologies 4087 S)
- DyLight-488 Lectin (Vector DL-1174)

### CLARITY restaining

Following imaging, samples were placed in PBST overnight to remove refractive index matching solution. Samples were then placed back in SDS clearing solution and incubated in a 60 °C oven for 4–6 days, depending on sample size and antibody binding strength. Antibody dissociation was verified by confocal microscopy set at high laser power, as well as by restaining samples using only secondary probes. Additional rounds of staining followed the procedure described above.

### Imaging of CLARITY samples

To prepare samples for imaging, samples were incubated in a refractive index matching solution (such as RapiClear or Histodenz) for 1–24 hours depending on sample size. Samples were mounted as previously described^8, 16^. Samples were imaged using an Olympus FV1200 confocal microscope system running Fluoview software, using a 10x, 0.6NA water immersion Olympus objective at 5 μm z-step resolution. Single photon excitation was used at the indicated wavelengths. Entire samples were obtained by mosaic tiling during imaging, reconstructed using default microscope software, and viewed in Imaris 8 (Bitplane).

### Streptozotocin injection

Diabetes was induced in adult (P42) and juvenile (P3) mice with a single intraperitoneal injection of streptozotocin (STZ, Sigma) dissolved in sodium citrate buffer of varying dosage (0, 100, 200, or 500 mg/kg). Animals were randomized to drug or vehicle control within cage groups. Animals were perfused at P6/P45, three days post-injection, or P15, twelve days post injection. Blood glucose levels were measured before animals were euthanized with a drop of blood from the tail and a glucometer (Contour, Bayer) by an experimenter blind to injection condition.

### Computational pancreas analysis

To quantify islet size and neural crest/innervation neighborhood values from volumetric images, we generated Python code to process images and extract relevant features in a semi-automated manner. First, we loaded individual imaging channels corresponding to neural crest/nerves and insulin. Datasets of human and whole adult rodent pancreas were downsampled by 0.5 with the Scipy Python package using bilinear interpretation, as the samples were too large to process at original resolution^67^. All sample images were normalized to the same intensity scale to account for staining and expression variability, and the pancreas was manually selected for analysis to exclude surrounding tissue such as intestine or spleen (as observed in Fig. 2a).

Samples were maximum intensity projected into 150 μm sections, corresponding to three standard deviations beyond mean islet size, improving signal to noise. In order to segment the section into identified islets, each section was intensity-thresholded using Otsu’s method from the Scikit-image (Skimage) Python package^68, 69^. Using the Scipy Python package, segmented regions were eroded and dilated for noise removal. Individual regions were identified using the Scipy multidimensional image processing package. Region properties, including the center of mass coordinates and size, were measured using the Skimage package. Unique positive regions of at least 5 pixels – corresponding to 124 μm^2^ in size, approximately the size of an endocrine cell^52, 53^ – were identified as islets. For each detected islet, a maximum intensity projection of the islet was created of the CLARITY slice in which the islet was detected and a bounding box of 20 pixels – or 100 μm – beyond the islet in each lateral direction. In this maximum intensity projection, an Otsu threshold was applied to identify the islet. Dilation and erosion were applied to remove noise.

Region properties were measured using the Skimage Python package, and an ellipse and convex hull was fit to each islet. The islet radius was estimated by assuming the convex hull approximated a circle. Each islet was assigned an islet neighborhood that was offset outward from the islet convex hull by an additional two pixels, or 10 μm. Positive neural crest/nerve pixels in the islet neighborhood were identified by applying an adaptive threshold from the OpenCV image processing Python package and eroding each region by one pixel – or 5 μm – to reduce noise^70^. The number of positive neural crest pixels in the islet neighborhood was counted, and islet-nerve interaction was calculated as the number of positive pixels divided by the total size of the islet. To remove artifacts, we excluded samples where either the percentage of positive nerve pixels or an automated entropy score from Skimage was greater than three standard deviations from the mean value in a test dataset, as these corresponded to non-neural features. Exclusion thresholds were held constant across all samples.

Code availability: All software was custom developed in Python using open-source libraries and are freely available upon request. Source code is available at: https://github.com/vburns88/ClinicalClarity. All CLARITY resources are freely available at www.clarityresourcecenter.org.

### Measurement simulations

For values of N ranging from 10 to 150, N random islets from an entire sample were selected, with replacement, and corresponding values for islet radius and NC/islet interaction were determined. Simulations were performed 100 times for each data point. Results were reported significant if the mean measured value differed (p < 0.05) between conditions using a two-tailed Student’s t-test.

### 2D and 3D comparisons

To provide an accurate comparison of slice and volumetric histology, the islet processing code as described above was run on eight evenly spaced 40 μm optical sections from a 1 mm CLARITY volume, representing every third 40 μm section from the organ. The number of islets was multiplied by three to scale the sections to the entire sample volume. For each islet detected in the 40 μm optical sections, a volumetric analysis was performed using the same region size in x and y, but extending in z to three standard deviations from the mean islet radius, representing the additional information supplied by a volumetric approach. Islet characteristics were quantified for the 2D and 3D models of the islet. Additional islet analysis was performed in 5 μm, 10 μm, 20 μm, 80 μm, and 110 μm optical sections in an identical manner. The islet size underestimation factor was calculated by subtracting the 2D radius from the 3D radius and dividing by the 3D radius for each of the optical sections.

### Statistics

Group mean comparisons were evaluated using two-tailed Student’s t-test. Non-normally distributed population comparisons (i.e. islet size distribution) were performed with a Kolmogorov–Smirnov test. All statistics were performed in Graphpad Prism. Effect sizes and variations were estimated from preliminary data to determine sample sizes.

## Electronic supplementary material

Supplementary data

## References

[1] Buesa RJ (2010). Staffing benchmarks for histology laboratories. Annals of diagnostic pathology.

[2] Tadrous PJ (2000). Methods for imaging the structure and function of living tissues and cells: 1. Optical coherence tomography. The Journal of pathology.

[3] Knowles CH, Lindberg G, Panza E, De Giorgio R (2013). New perspectives in the diagnosis and management of enteric neuropathies. *Nature reviews*. Gastroenterology & hepatology.

[4] Grone E (2014). Reduced intraepidermal nerve fiber density in patients with chronic ischemic pain in peripheral arterial disease. Pain.

[5] Ziegler D (2014). Early detection of nerve fiber loss by corneal confocal microscopy and skin biopsy in recently diagnosed type 2 diabetes. Diabetes.

[6] Tainaka K (2014). Whole-body imaging with single-cell resolution by tissue decolorization. Cell.

[7] Tang S-C, Chiu Y-C, Hsu C-T, Peng S-J, Fu Y-Y (2013). Plasticity of Schwann cells and pericytes in response to islet injury in mice. Diabetologia.

[8] Chung K (2013). Structural and molecular interrogation of intact biological systems. Nature.

[9] Murray E (2015). Simple, Scalable Proteomic Imaging for High-Dimensional Profiling of Intact Systems. Cell.

[10] Renier N (2014). iDISCO: a simple, rapid method to immunolabel large tissue samples for volume imaging. Cell.

[11] Susaki EA (2015). Advanced CUBIC protocols for whole-brain and whole-body clearing and imaging. Nature protocols.

[12] Pan C (2016). Shrinkage-mediated imaging of entire organs and organisms using uDISCO. Nat Methods.

[13] Richardson DS, Lichtman JW (2015). Clarifying Tissue Clearing. Cell.

[14] Treweek JB, Gradinaru V (2016). Extracting structural and functional features of widely distributed biological circuits with single cell resolution via tissue clearing and delivery vectors. Curr Opin Biotechnol.

[15] Sylwestrak EL, Rajasethupathy P, Wright MA, Jaffe A, Deisseroth K (2016). Multiplexed Intact-Tissue Transcriptional Analysis at Cellular Resolution. Cell.

[16] Tomer R, Ye L, Hsueh B, Deisseroth K (2014). Advanced CLARITY for rapid and high-resolution imaging of intact tissues. Nature protocols.

[17] Livet J (2007). Transgenic strategies for combinatorial expression of fluorescent proteins in the nervous system. Nature.

[18] Muzumdar MD, Tasic B, Miyamichi K, Li L, Luo L (2007). A global double-fluorescent Cre reporter mouse. Genesis.

[19] Treweek JB (2015). Whole-body tissue stabilization and selective extractions via tissue-hydrogel hybrids for high-resolution intact circuit mapping and phenotyping. Nature protocols.

[20] Yang B (2014). Single-cell phenotyping within transparent intact tissue through whole-body clearing. Cell.

[21] Ye, L. *et al*. Wiring and Molecular Features of Prefrontal Ensembles Representing Distinct Experiences. *Cell*, doi:10.1016/j.cell.2016.05.010 (2016). 10.1016/j.cell.2016.05.010 PMC5708551 27238022

[22] Nekrep N, Wang J, Miyatsuka T, German MS (2008). Signals from the neural crest regulate beta-cell mass in the pancreas. Development.

[23] Kozlova EN, Jansson L (2009). Differentiation and migration of neural crest stem cells are stimulated by pancreatic islets. Neuroreport.

[24] Plank JL (2011). Influence and timing of arrival of murine neural crest on pancreatic beta cell development and maturation. Developmental biology.

[25] Shimada K, Tachibana T, Fujimoto K, Sasaki T, Okabe M (2012). Temporal and Spatial Cellular Distribution of Neural Crest Derivatives and Alpha Cells during Islet Development. Acta histochemica et cytochemica.

[26] Borden P, Houtz J, Leach SD, Kuruvilla R (2013). Sympathetic innervation during development is necessary for pancreatic islet architecture and functional maturation. Cell reports.

[27] Reinert RB (2014). Vascular endothelial growth factor coordinates islet innervation via vascular scaffolding. Development.

[28] Donev SR (1984). Ultrastructural evidence for the presence of a glial sheath investing the islets of Langerhans in the pancreas of mammals. Cell and tissue research.

[29] Scaglia L, Cahill CJ, Finegood DT, Bonner-Weir S (1997). Apoptosis Participates in the Remodeling of the Endocrine Pancreas in the Neonatal Rat 1. Endocrinology.

[30] Dhawan S, Georgia S, Bhushan A (2007). Formation and regeneration of the endocrine pancreas. Current opinion in cell biology.

[31] Benthem L, Mundinger TO, Taborsky GJ (2001). Parasympathetic inhibition of sympathetic neural activity to the pancreas. Am J Physiol Endocrinol Metab.

[32] Abdulreda MH (2011). High-resolution, noninvasive longitudinal live imaging of immune responses. Proceedings of the National Academy of Sciences of the United States of America.

[33] Rodriguez-Diaz R (2011). Innervation patterns of autonomic axons in the human endocrine pancreas. Cell metabolism.

[34] Alanentalo T (2007). Tomographic molecular imaging and 3D quantification within adult mouse organs. Nat Methods.

[35] Nyman LR (2008). Real-time, multidimensional *in vivo* imaging used to investigate blood flow in mouse pancreatic islets. J Clin Invest.

[36] Dorsky RI, Moon RT, Raible DW (1998). Control of neural crest cell fate by the Wnt signalling pathway. Nature.

[37] Teitelman G, Guz Y, Ivkovic S, Ehrlich M (1998). Islet injury induces neurotrophin expression in pancreatic cells and reactive gliosis of peri-islet Schwann cells. Journal of neurobiology.

[38] Georgia S, Bhushan Aβ (2004). cell replication is the primary mechanism for maintaining postnatal β cell mass. The Journal of clinical investigation.

[39] Kushner JA (2005). Cyclins D2 and D1 are essential for postnatal pancreatic β-cell growth. Molecular and cellular biology.

[40] Gregg BE (2012). Formation of a human beta-cell population within pancreatic islets is set early in life. J Clin Endocrinol Metab.

[41] Amella C (2008). Spatial and temporal dynamics of innervation during the development of fetal human pancreas. Neuroscience.

[42] Mierke CT (2014). The fundamental role of mechanical properties in the progression of cancer disease and inflammation. Reports on progress in physics. Physical Society.

[43] Himsworth HP (1949). The syndrome of diabetes mellitus and its causes. Lancet.

[44] Jo J, Choi MY, Koh D-S (2007). Size distribution of mouse Langerhans islets. Biophysical journal.

[45] Cheng L (1999). Tumor size predicts the survival of patients with pathologic stage T2 bladder carcinoma: a critical evaluation of the depth of muscle invasion. Cancer.

[46] Edge, S. B. & American Joint Committee on Cancer. *AJCC cancer staging manual*. 7th edn, (Springer, 2010). 10.1245/s10434-010-0985-4 20180029

[47] Silverberg SG (1976). Reproducibility of the mitosis count in the histologic diagnosis of smooth muscle tumors of the uterus. Human pathology.

[48] Jannink I, Risberg B, Van Diest PJ, Baak JP (1996). Heterogeneity of mitotic activity in breast cancer. Histopathology.

[49] Prasad ML, Osborne MP, Giri DD, Hoda SA (2000). Microinvasive carcinoma (T1mic) of the breast: clinicopathologic profile of 21 cases. The American journal of surgical pathology.

[50] Ploussard G (2010). Pathological findings and prostate specific antigen outcomes after radical prostatectomy in men eligible for active surveillance–does the risk of misclassification vary according to biopsy criteria?. The Journal of urology.

[51] Boehringer A, Adam P, Schnabl S, Hafner HM, Breuninger H (2015). Analysis of incomplete excisions of basal-cell carcinomas after breadloaf microscopy compared with 3D-microscopy: a prospective randomized and blinded study. Journal of cutaneous pathology.

[52] Kim A (2009). Islet architecture: A comparative study. Islets.

[53] Kilimnik G, Jo J, Periwal V, Zielinski MC, Hara M (2012). Quantification of islet size and architecture. Islets.

[54] Cormack, D. H. *Essential histology*. 2nd edn, (Lippincott Williams & Wilkins, 2001).

[55] Weaver DL (2010). Pathology evaluation of sentinel lymph nodes in breast cancer: protocol recommendations and rationale. Modern pathology: an official journal of the United States and Canadian Academy of Pathology, Inc.

[56] Mabry H, Giuliano AE (2007). Sentinel node mapping for breast cancer: progress to date and prospects for the future. Surgical oncology clinics of North America.

[57] Cheng L, Weaver AL, Bostwick DG (2000). Predicting extravesical extension of bladder carcinoma: a novel method based on micrometer measurement of the depth of invasion in transurethral resection specimens. Urology.

[58] Boehringer A (2011). Extramammary Paget’s disease: extended subclinical growth detected using three-dimensional histology in routine paraffin procedure and course of the disease. Dermatologic surgery: official publication for American Society for Dermatologic Surgery [et al.].

[59] Batchelor TT (2013). Improved tumor oxygenation and survival in glioblastoma patients who show increased blood perfusion after cediranib and chemoradiation. Proceedings of the National Academy of Sciences of the United States of America.

[60] Scott GD, Blum ED, Fryer AD, Jacoby DB (2014). Tissue optical clearing, three-dimensional imaging, and computer morphometry in whole mouse lungs and human airways. American journal of respiratory cell and molecular biology.

[61] Torres R, Vesuna S, Levene MJ (2014). High-resolution, 2- and 3-dimensional imaging of uncut, unembedded tissue biopsy samples. Archives of pathology & laboratory medicine.

[62] Belle M (2017). Tridimensional Visualization and Analysis of Early Human Development. Cell.

[63] Renier N (2016). Mapping of Brain Activity by Automated Volume Analysis of Immediate Early Genes. Cell.

[64] McCann MT, Ozolek JA, Castro CA, Parvin B, Kovacevic J (2015). Automated Histology Analysis [Opportunities for signal processing]. Ieee Signal Proc Mag.

[65] Magee D (2015). Histopathology in 3D: From three-dimensional reconstruction to multi-stain and multi-modal analysis. J Pathol Inform.

[66] Esteva A (2017). Dermatologist-level classification of skin cancer with deep neural networks. Nature.

[67] Jones E, O. E., Peterson, P. *et al*. *SciPy: Open Source Scientific Tools for Python*, http://www.scipy.org/ (2001).

[68] Otsu N (1979). Threshold Selection Method from Gray-Level Histograms. Ieee T Syst Man Cyb.

[69] van der Walt, S. *et al*. scikit-image: image processing in Python. *Peerj* **2**, doi:ARTN e45310.7717/peerj.453 (2014). 10.7717/peerj.453 PMC4081273 25024921

[70] Bradski G (2000). The OpenCV library. Dr Dobbs J.

