# Supplementary material for: Pathways to clinical CLARITY: volumetric analysis of irregular, soft, and heterogeneous tissues in development and disease
Source: Sci Rep. 2017 Jul 19;7:5899. doi: 10.1038/s41598-017-05614-4 (PMC5517617; doi:10.1038/s41598-017-05614-4)
Supplement: Supplementary file 1 — Supplementary data [file 41598_2017_5614_MOESM1_ESM.pdf]

Supplemental Information

Pathways to clinical CLARITY: volumetric analysis of irregular, soft, and heterogeneous tissues in development and disease

Brian Hsueh*[1], Vanessa M. Burns*[2], Philip Pauerstein[3], Katherine Holzem[4], Li Ye[5,6], Kristin Engberg[1], Ai-Chi Wang[1], Xueying Gu[3], Harini Chakravarthy[3], H. Efsun Arda[3], Gregory Charville[7], Hannes Vogel[7], Igor R. Efimov[4,8], Seung Kim[3,6], and Karl Deisseroth[1,5,6]

[1]Department of Bioengineering, Stanford University
[2]Department of Chemical and Systems Biology, Stanford University
[3]Department of Developmental Biology, Stanford University
[4]Department of Biomedical Engineering, Washington University in St. Louis
[5]Department of Psychiatry and Behavioral Sciences, Stanford University
[6]Howard Hughes Medical Institute
[7]Department of Pathology, Stanford University
[8]Department of Biomedical Engineering, The George Washington University

Stanford University
Stanford, CA 94305 USA

Correspondence: K.D. (deissero@stanford.edu)

Figure S1

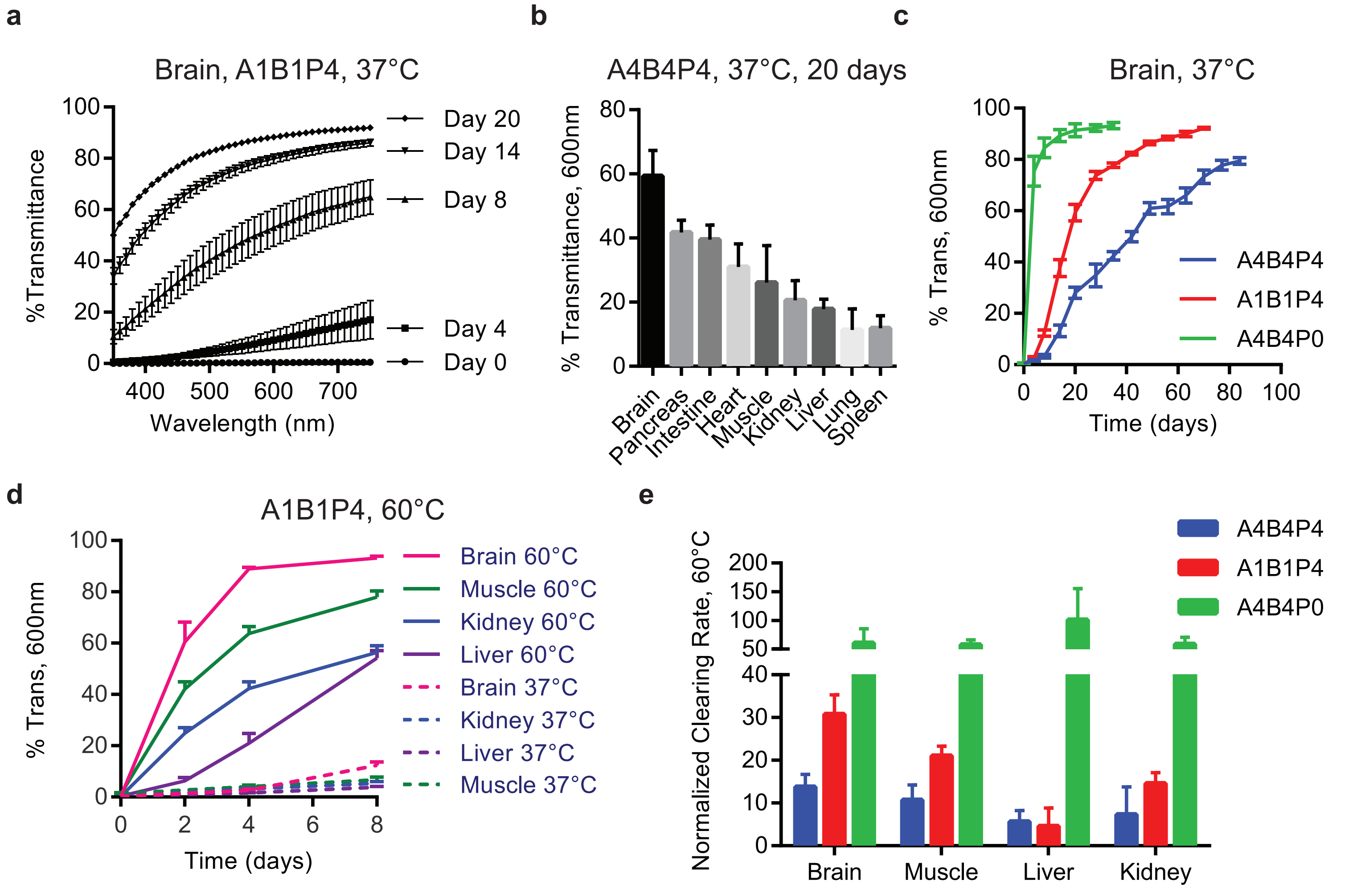
a
Brain, A1B1P4, 37°C
%Transmittance
Wavelength (nm)
Day 20
Day 14
Day 8
Day 4
Day 0
b
A4B4P4, 37°C, 20 days
% Transmittance, 600nm
Brain
Pancreas
Intestine
Heart
Muscle
Kidney
Liver
Lung
Spleen
c
Brain, 37°C
% Trans, 600nm
Time (days)
A4B4P4
A1B1P4
A4B4P0
d
A1B1P4, 60°C
% Trans, 600nm
Time (days)
Brain 60°C
Muscle 60°C
Kidney 60°C
Liver 60°C
Brain 37°C
Kidney 37°C
Liver 37°C
Muscle 37°C
e
Normalized Clearing Rate, 60°C
A4B4P4
A1B1P4
A4B4P0
Brain
Muscle
Liver
Kidney

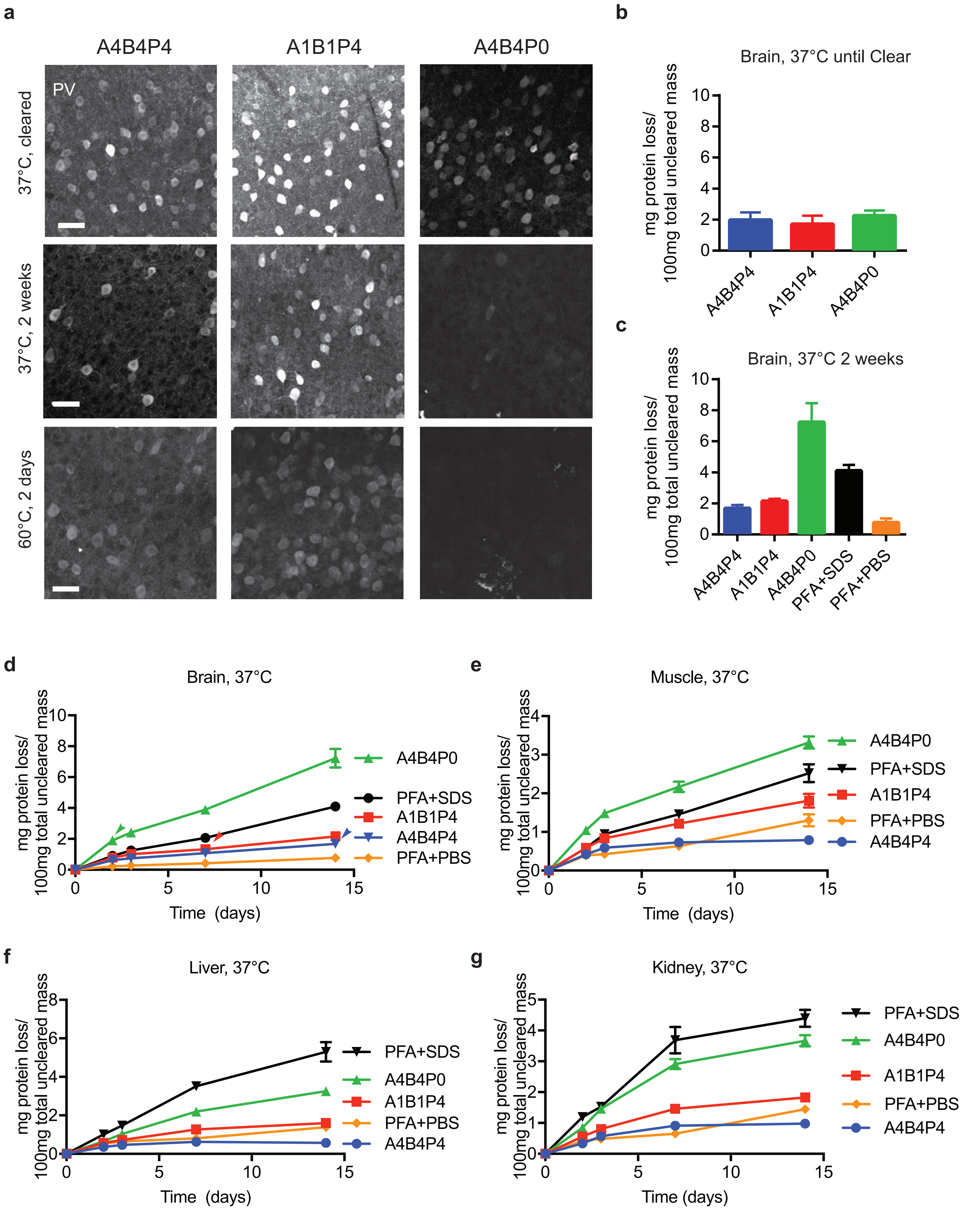
a
A4B4P4
A1B1P4
A4B4P0
PV
37°C, cleared
37°C, 2 weeks
60°C, 2 days
b
Brain, 37°C until Clear
mg protein loss/
100mg total uncleared mass
A4B4P4
A1B1P4
A4B4P0
c
Brain, 37°C 2 weeks
A4B4P4
A1B1P4
A4B4P0
PFA+SDS
PFA+PBS
d
Brain, 37°C
Time (days)
e
Muscle, 37°C
f
Liver, 37°C
g
Kidney, 37°C

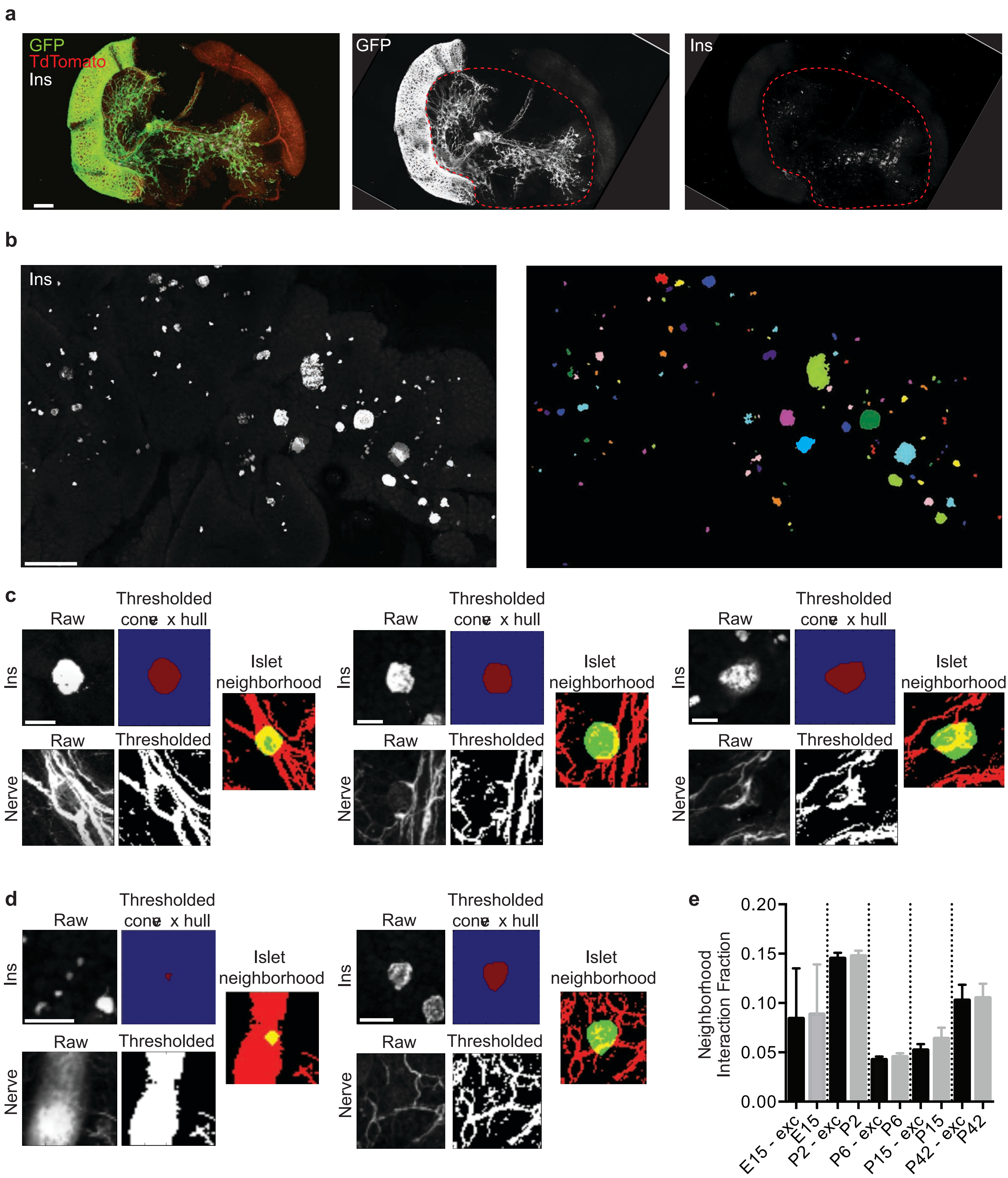
a
GFP
TdTomato
Ins
GFP
Ins
b
Ins
c
Raw
Thresholded
convex hull
Ins
Raw
Thresholded
Nerve
Islet
neighborhood
d
Raw
Thresholded
convex hull
Ins
Raw
Thresholded
Nerve
Islet
neighborhood
e
Neighborhood
Interaction Fraction
0.20
0.15
0.10
0.05
0.00
E15 - exc
E15
P2 - exc
P2
P6 - exc
P6
P15 - exc
P15
P42 - exc
P42

Figure S4

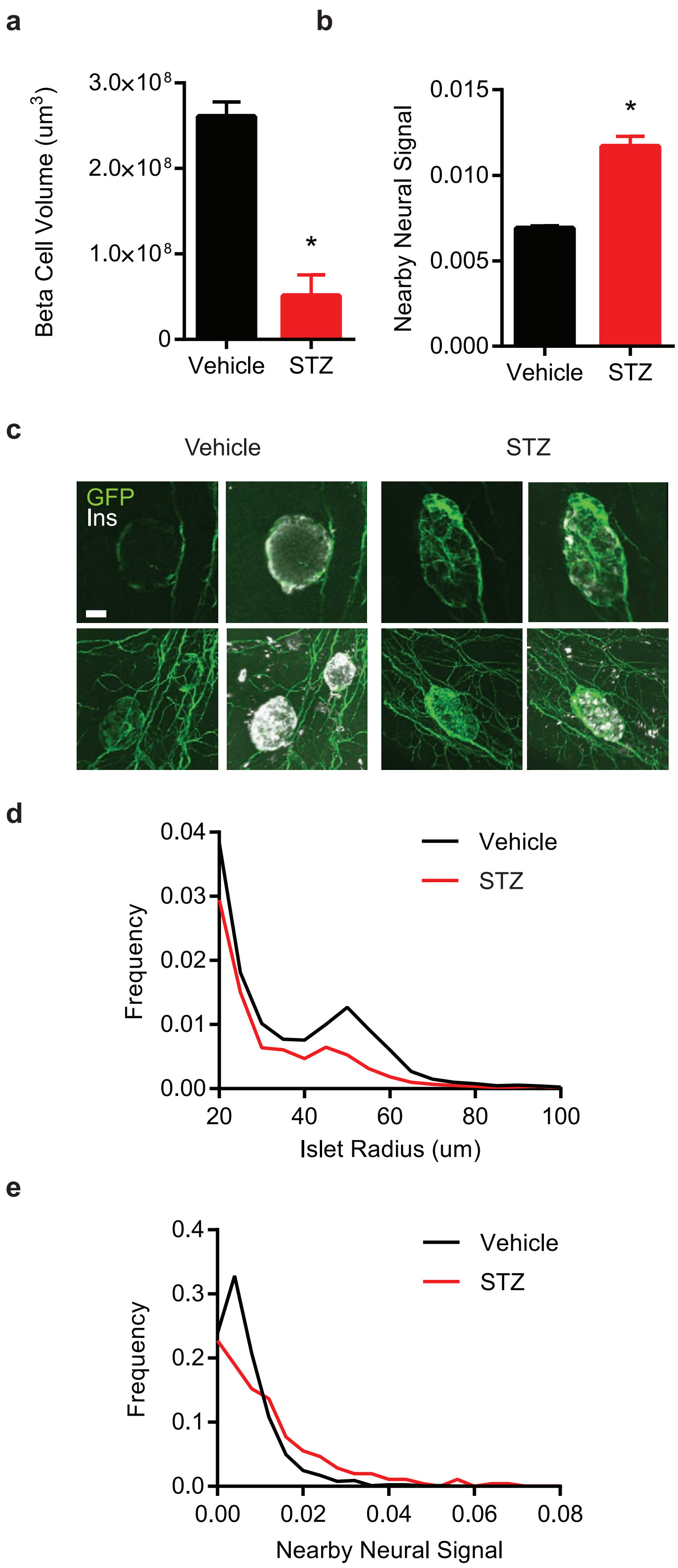
a
b
3.0x10⁸
2.0x10⁸
1.0x10⁸
0
Beta Cell Volume (um³)
*
Vehicle
STZ
0.015
0.010
0.005
0.000
Nearby Neural Signal
*
Vehicle
STZ
c
Vehicle
STZ
GFP
Ins
d
0.04
0.03
0.02
0.01
0.00
Frequency
Vehicle
STZ
20
40
60
80
100
Islet Radius (um)
e
0.4
0.3
0.2
0.1
0.0
Frequency
Vehicle
STZ
0.00
0.02
0.04
0.06
0.08
Nearby Neural Signal

Figure S5

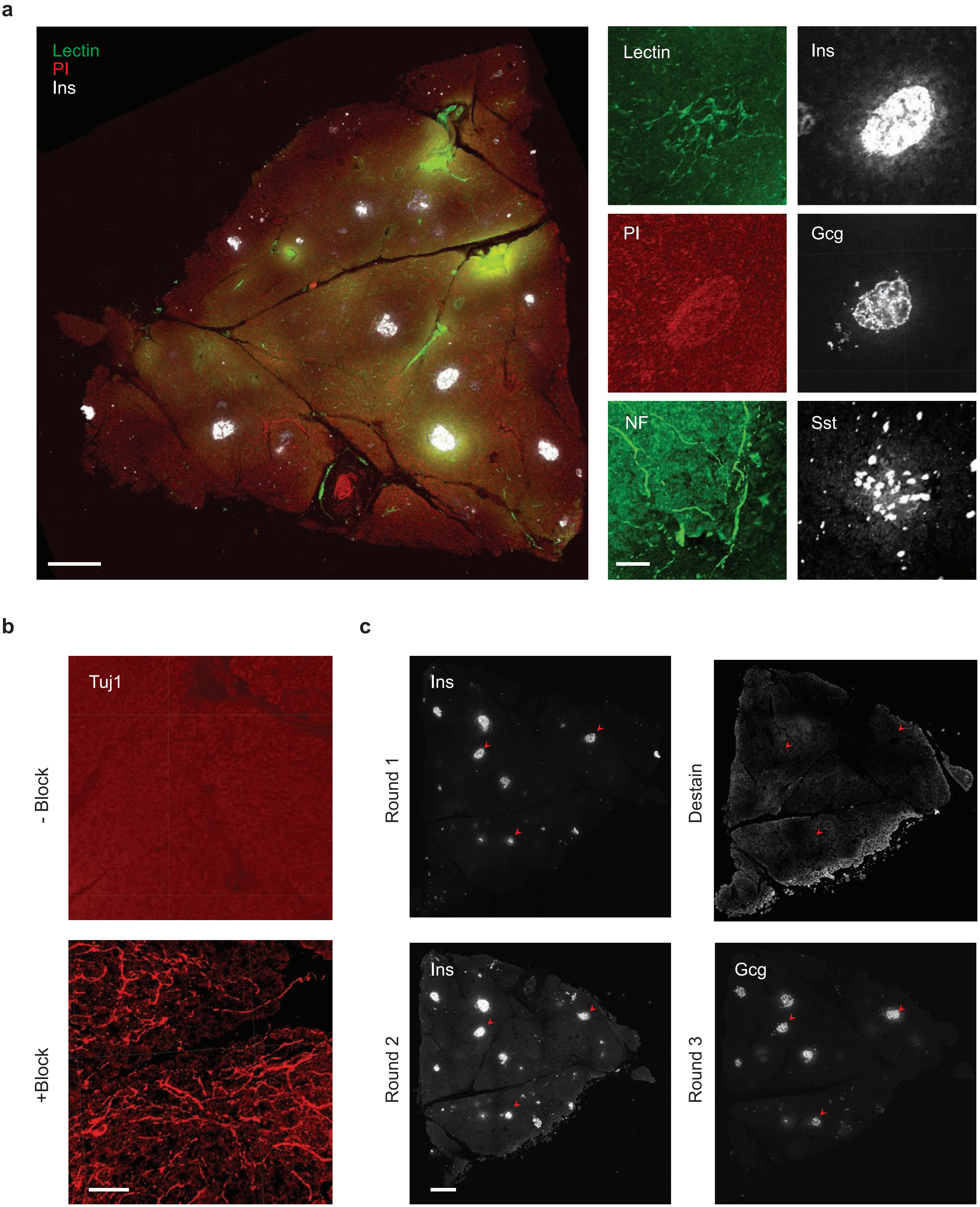
a
Lectin
PI
Ins
Lectin
Ins
PI
Gcg
NF
Sst
b
c
Tuj1
- Block
+Block
Ins
Round 1
Destain
Ins
Round 2
Gcg
Round 3

Figure S6

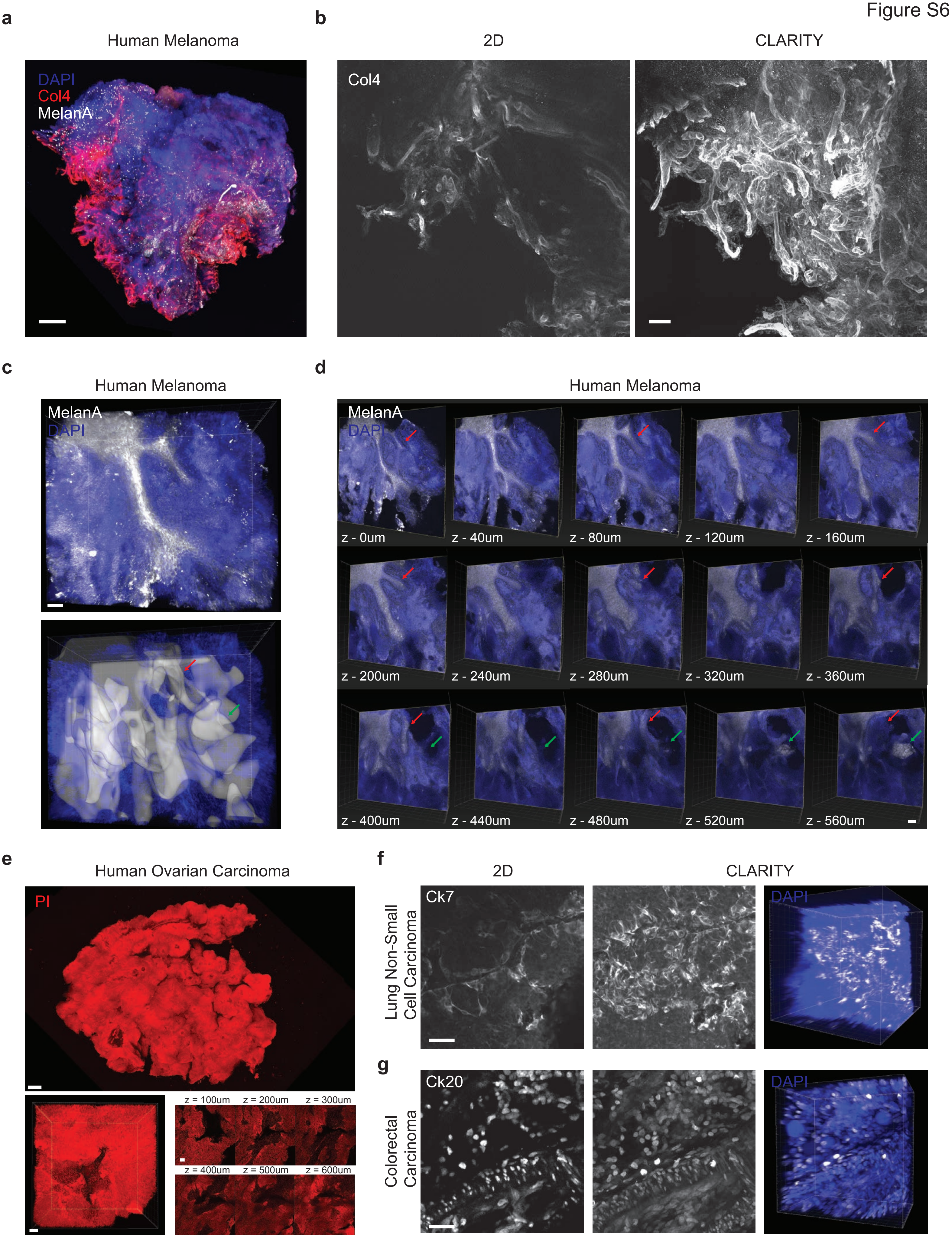

a
Human Melanoma
DAPI
Col4
MelanA
b
2D
CLARITY
Col4
c
Human Melanoma
MelanA
DAPI
d
Human Melanoma
MelanA
DAPI
z - 0um
z - 40um
z - 80um
z - 120um
z - 160um
z - 200um
z - 240um
z - 280um
z - 320um
z - 360um
z - 400um
z - 440um
z - 480um
z - 520um
z - 560um
e
Human Ovarian Carcinoma
PI
z = 100um
z = 200um
z = 300um
z = 400um
z = 500um
z = 600um
f
2D
CLARITY
Lung Non-Small Cell Carcinoma
Ck7
DAPI
g
Colorectal Carcinoma
Ck20
DAPI

a

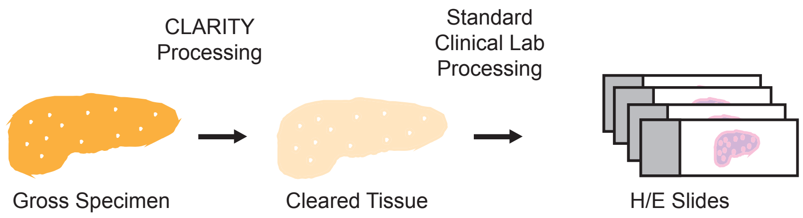
CLARITY
Processing
Standard
Clinical Lab
Processing
Gross Specimen
Cleared Tissue
H/E Slides

b

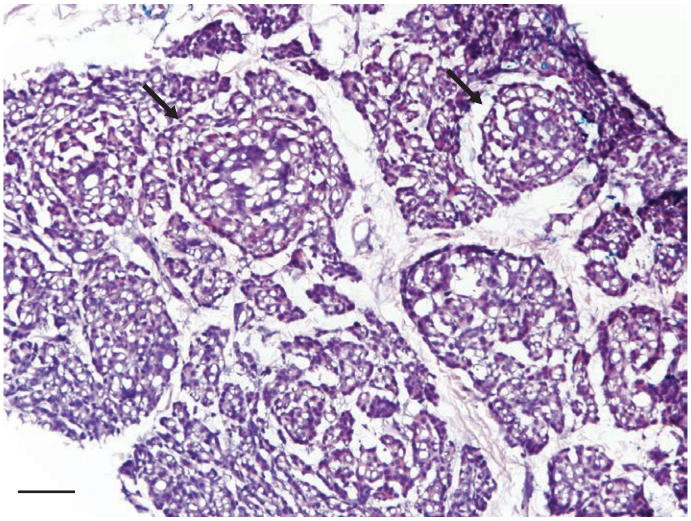

Supplemental Figure Legends

Figure S1: Optical properties of CLARITY gels in diverse organ systems. (a) Clearing kinetics were determined using UV-spectrophotometer measurements taken over time, here illustrated using brain tissue fixed with A1B1P4 CLARITY gel and cleared at 37°C. Under all clearing conditions, transmission of longer wavelength light is more efficient than shorter wavelength light, with implications for optimal fluorophore selection. n = 6 2 mm sections. (b) When cleared under identical conditions, an array of different tissue types results in vastly different levels of optical transparency due to organ-organ heterogeneity. n = 6 2 mm sections. (c) 2 mm slices of brain tissue fixed with different hydrogel formulations were cleared and measured for transparency at different time points. Clearing rates were computed by fitting clearing curves to the first order transformation reaction kinetics equation $y = Y_{max} (1 - e^{-kt})$ and normalized to Brain A4B4P4. n = 6 2 mm sections. (d) Rates of tissue clearing were increased for all tissue types when clearing was performed at 60°C, illustrated here with several tissue types embedded in A1B1P4 CLARITY gel. n = 6 2 mm sections. (e) Summary of clearing rates for different tissue types when cleared at 60°C. A1B1P4 CLARITY tissues can be cleared 15-30x faster at 60°C, A4B4P0 CLARITY tissues 50x faster or more when compared to A4B4P4 cleared at 37°C. n = 6 2 mm sections.

Figure S2: Protein retention properties of CLARITY gels in diverse organ systems.
(a) When removed from clearing solution once the tissue was clear, all gel types retained protein effectively and were suitable for detection using antibody probes, illustrated here with parvalbumin (PV) antibody incubated for 48 hours. However, prolonged exposure to clearing solution can result in catastrophic protein loss at both 37°C and 60°C for tissues fixed with A4B4P0 gel. Images were acquired with identical microscope power settings and are maximum intensity projections over 1 mm. Scale bar = 50 µm. (b) Quantification of protein loss in tissues immediately after clearing. n = 6. (c) Quantification of protein loss in tissues after 2 weeks' excess in clearing solution at 37°C. A4B4P0 gel lost excessive protein, even compared to PFA fixed tissue in SDS. n = 6. (d-g) Quantification of protein loss in brain, muscle, liver, and kidney tissue over time while cleared at 37°C. Arrowheads indicate when brain tissue is optically cleared; those values are used in panel (b). n = 6.

Figure S3: Computational processing and analysis pipeline. (a) Raw image stacks (left) for each sample were loaded and split into individual channels. The pancreas (red dotted line) was manually selected for analysis if extra tissue was present. Scale bar = 500 µm. (b) After normalization and subsectioning the volume into 150 µm maximum intensity projections (left, raw image), the sample was thresholded. Erosion and morphological closing generated unique regions, represented by individual colors (right), which could be analyzed for physical properties, including position and size. For each detected islet, a convex hull was fit to positive pixels. Objects was found to be fewer than 5 pixels in area (representing 124 μm^2 in area, less than a cell), were excluded from analysis. An ellipse was fit to each hull to determine the major and minor axes. The hull was offset by two pixels (10 µm) toward the outer dimension to generate an islet neighborhood. Scale bar: 250 µm. (c) Example raw signals from the islet and neural channel are shown with thresholded, positive neural pixels and fitted convex hull islet, followed by the islet neighborhood (green) overlayed on the thresholded neural channel (red). Three example islets are shown. Scale bars: 50 µm. (d) Processing automatically excludes non-

neural features (Methods). Scale bar: 50 μm. (e) Example means for islet-neural interactions in each islet neighborhood for datasets with automatically excluded islets (black) or all detected islets (grey) across ages. No differences were observed due to automatic exclusion parameters.

Figure S4: Neuroendocrine pancreas in disease. CLARITY tools and automated analysis were sufficient for detecting organ-wide changes in tissue architecture in an established model of drug-induced diabetes. (a-b) 200mg/kg Streptozotocin (STZ) treatment of adult Wnt1-Cre x Rosa26-mT/mG mice resulted in reduction in beta cell volume (A, $p<0.05$, unpaired t-test), and an increase in surrounding neural-crest derived (glial or neural) structures (B, $p<0.05$, unpaired t-test) three days following acute exposure. n = 3 pancreata per condition, error bars are s.e.m. (c) Representative 2D (top) and 3D (bottom) images of GFP+ structures alone (left) or with Ins (right) show islets with increased neural structures following STZ injection. Scale bar = 50 μm. (d, e) Population distributions across all islets in both STZ and vehicle-treated mice show a reduced fraction of large islets and general increase in peri-islet glial or neural structures following STZ injection as detected by the automated software pipeline.

Figure S5: CLARITY in fresh frozen human specimens. (a) CLARITY processing was compatible with human specimens that are flash frozen immediately following resection, as commonly performed in surgical settings, and further was compatible with staining with both small molecule dyes (Lectin, Propidium Iodide (PI)) and antibody probes (Neurofilament (NF), Insulin (Ins), Glucagon (Gcg), and Somatostatin (Sst)). This 200 μm-thick frozen section from a healthy adult pancreas was obtained from a human tissue bank, embedded in A1B1P4 hydrogel, and cleared at 60°C for 2 days. Scale bar = 500 μm (low magnification), 100 μm (high magnification of different islets for each stain). (b) Unlike CLARITY samples processed following PFA fixation, we found that fresh frozen specimens require blocking with serum prior to antibody staining to reduce background binding. Scale bar = 100 μm. (c) Frozen specimens processed with CLARITY could be stained through multiple rounds of antibody labeling and removal. Arrows indicate identical islets. Staining was performed with 3-6 hour antibody incubations, and antibodies were removed through clearing at 60°C for 24 hours. Scale bar = 500 μm.

Figure S6: CLARITY in human cancer specimens. (a) CLARITY enabled clearing, dye and antibody labeling, and imaging of pathological samples obtained from a tissue bank, such as this frozen melanoma biopsy, embedded in A1B1P4 hydrogel, cleared for 2 weeks at 37°C, labeled with DAPI, Col4, and melanA, and imaged on a confocal microscope. Scale bar = 500 μm (b) When compared with a 2D optical section, CLARITY enables visualization of complex tissue architecture that may be diagnostically relevant, such as vasculature labelled with Col4. Scale bar = 50 μm. (c, d) CLARITY enables visualization of complex 3-dimensional tumor boundaries, including structures that change drastically within serial sections, such as appendages or offshoots (arrows), which can be assessed as serial sections or through manually-annotated volumetric rendering with viewing software such as Imaris. Scale bar = 100 μm. (e-g) Multiple human tumor types can be cleared and visualized in 3D utilizing CLARITY, enabling visualization of glandular or tubular structures (e.g. in ovarian cancer), and tumor specific diagnostic markers such as Cytokeratin 7 (Ck7, in lung non-small cell carcinoma) and Cytokeratin 20 (Ck20, in colorectal carcinoma). Scale bars: 500 μm (e, low magnification), 100 μm (e, high magnification), 50 μm (f, g).

Figure S7: Post-CLARITY pathological processing. (a) Schematic demonstrating post-CLARITY workflow. After a human pancreas sample (from Fig. 4) was cleared, stained, and imaged using a biphasic CLARITY protocol, the sample was stored in PBST. The sample was then submitted to a clinical pathology lab for frozen section processing and hematoxylin/eosin staining. (b) Hematoxylin/eosin labeling of post-CLARITY tissue reliably labels intact structures such as islets (arrows). Images were taken with a light microscope under 10x magnification. Scale bar = 50 µm.

Hsueh, Burns, et al., 2017
Detailed Protocol for Biphasic CLARITY

Introduction:
Published CLARITY protocols[1-3], designed for solid organs with fixed architectures such as the brain, result in the formation of a solid-phase gel on exterior surfaces of the tissue specimen which must subsequently be mechanically removed, potentially damaging fine structures. Furthermore, this rigid gel results in deleterious clumping, shearing, and expansion within tissue cavities, which is particularly harmful for soft and irregular tissue samples. We formulated a two-phase hydrogel consisting of a solid gel only within the tissue parenchyma itself but liquid elsewhere, including at irregular tissue and cavity boundaries. This enables fragile tissues to achieve mechanical and chemical stability – including clinical biopsies and mouse embryos – without damage incurred from solid gel formation during the embedding process.

In addition to considerations made for soft and irregular tissues, this protocol is directly compatible with any fixed tissue sample, without the need for initial hydrogel-based perfusion or fixation, thereby making it particularly suitable for use with fixed clinical samples.

Nomenclature:
Hydrogel formulations (e.g. A4B4P4) are reported as proportions of key chemical components Acrylamide (A), Bis-Acrylamide (B), and Paraformaldehyde (P) relative to original concentrations published in Chung et al.[1]

Acrylamide polymer mixtures can either form solid hydrogels or remain in liquid phase depending on the concentration of polymer components. Here we report the material phase of the hydrogel as solid or liquid when polymerized directly from solution. Liquid hydrogels will take on a solid phase within the tissue only.

<u>A4B4P4 Hydrogel Solution</u> (Solid Phase when polymerized):

Chemical	Volume in 400 mL	Final Concentration (%)
Acrylamide (40%)	40 mL	4%
Bis-Acrylamide (2%)	10 mL	.05%
Paraformaldehyde (16%)	100 mL	4%
PBS (10x)	40 mL	1x
VA-044 Initiator	1 g	.25%
dH_2O	210 mL	-

Supplemental Table 1: A4B4P4 Hydrogel Solution. This hydrogel forms a solid phase when polymerized.

<u>A4B4P0 Hydrogel Solution</u> (Solid Phase when polymerized):

Chemical	Volume in 400 mL	Final Concentration (%)
Acrylamide (40%)	40 mL	4%
Bis-Acrylamide (2%)	10 mL	.05%
Paraformaldehyde (16%)	-	-
PBS (10x)	40 mL	1x
VA-044 Initiator	1 g	.25%
dH_2O	310 mL	-

Supplemental Table 2: A4B4P0 Hydrogel Solution. This hydrogel forms a solid phase when polymerized.

<u>A1B1P4 Hydrogel Solution</u> (Liquid Phase when polymerized):

Chemical	Volume in 400 mL	Final Concentration (%)
Acrylamide (40%)	10 mL	1%
Bis-Acrylamide (2%)	2.5 mL	.0125%
Paraformaldehyde (16%)	100 mL	4%
PBS (10x)	40 mL	1x
VA-044 Initiator	1 g	.25%
dH_2O	247.5 mL	-

Supplemental Table 3: A1B1P4 Hydrogel Solution. This hydrogel forms a liquid phase when polymerized.

<u>A2B2P4 Hydrogel Solution</u> (Liquid Phase when polymerized):

Chemical	Volume in 400 mL	Final Concentration (%)
Acrylamide (40%)	20 mL	2%
Bis-Acrylamide (2%)	5 mL	.025%
Paraformaldehyde (16%)	100 mL	4%
PBS (10x)	40 mL	1x
VA-044 Initiator	1 g	.25%
dH_2O	235 mL	-

Supplemental Table 4: A2B2P4 Hydrogel Solution. This hydrogel forms a liquid phase when polymerized.

<u>A4B0P4 Hydrogel Solution</u> (Liquid Phase when polymerized):

Chemical	Volume in 400 mL	Final Concentration (%)
Acrylamide (40%)	40 mL	4%
Bis-Acrylamide (2%)	-	-
Paraformaldehyde (16%)	100 mL	4%
PBS (10x)	40 mL	1x
VA-044 Initiator	1 g	.25%
dH_2O	220 mL	-

Supplemental Table 5: A4B0P4 Hydrogel Solution. This hydrogel forms a liquid phase when polymerized.

Biphasic CLARITY Protocol:

1. Fix tissue sample in cold 4% Paraformaldehyde (PFA) up to 12 hours overnight.

   For animal specimens, this can be achieved via perfusion of ice-cold PBS followed by perfusion of ice-cold 4% PFA or by direct immersion fixation.

   For fresh clinical specimens, this can be achieved via rinsing with ice-cold PBS followed by direct immersion fixation in ice-cold 4% PFA.

   For frozen specimens, samples can be thawed either in ice-cold 4% PFA or directly in CLARITY hydrogel solution.

   The volume of PFA should be in excess of sample size. For most applications, 50 mL in a falcon tube is sufficient.

   After PFA fixation, samples can be stored (i.e. in PBS or cryoprotectant and frozen) indefinitely.

2. (Optional) Perform any post-fixation processing of tissue as with standard histology, for example, decalcification of bony tissue or blocking into smaller pieces.

   Decalcification of mouse bones can be performed with incubation in 10-20% EDTA for 2-14 days depending on animal age and size of bone.

   Antigen retrieval may be appropriately performed at this point for some epitopes. For example, recovery of BrdU labeling of nuclear DNA requires a short incubation in 0.1M HCl.

3. Post-fix tissue sample in CLARITY hydrogel at 4°C.

   Gel formulation should be selected based on desired CLARITY properties (i.e. faster clearing speed vs. improved protein retention and multi-round staining).

   The length of incubation period will depend on sample size. We have found a general rule to be approximately 12 hours of incubation per 1000um sample thickness at a minimum (e.g. 1 mm-thick sections incubated for 12 hours, 6 mm-thick samples such as mouse brain incubated for 2-3 days).

   The volume of hydrogel should be in excess of sample size. For most applications, 50 mL in a falcon tube is sufficient.

4. If using Solid Phase CLARITY hydrogel, transfer sample to Liquid Phase polymerization solution immediately prior to polymerization.

Setting up a two-phase hydrogel during polymerization enables the formation of a rigid, solid hydrogel throughout the tissue parenchyma while keeping the surrounding solution in liquid phase for easy removal. Within the tissue, where there are high concentrations of proteins to serve as additional cross-linking agents, all CLARITY formulations will form solid structures.

Solid Phase CLARITY Formulations: A4B4P4, A4B4P0
Liquid Phase polymerization solution: A1B1P4, A4B0P4, A4B0P0, PBS

As the Liquid Phase solution is only transient, we have found that the selection of the specific formulation used to be a matter of user preference, and Solid Phase polymerization is robust even when performed in PBS. We recommend maximizing similarity between Solid Phase and Liquid Phase for each individual sample: i.e. using A4B0P4 for A4B4P4 gels, and A4B0P0 for A4B4P0 gels.

5. If using Liquid Phase CLARITY hydrogel, keep sample in Liquid Phase CLARITY hydrogel.

    As the tissue contains high concentrations of proteins that will serve as additional cross-linkers to the hydrogel, the polymerized gel will naturally form a solid within the tissue parenchyma and remain a liquid otherwise.

6. Polymerize CLARITY Hydrogel to embed tissue specimen in polyacrylamide

    (Option 1): As described previously[1,3-5], polymerization can be performed by first degassing the solution under a vacuum, and flushing the solution with nitrogen gas in order to remove oxygen, an inhibitor of the polymerization reaction. Keep under vacuum conditions for 10 minutes before quickly recapping the tube.

    (Option 2): Polymerization can also be achieved through direct inhibition of oxygen via a thick layer of hydrophobic oil – such as Castor Oil (Fisher AC404165000) or Sunflower Oil (Fisher NC9967447) – on top of the liquid phase solution in the tube[6]. While simpler due to not needing vacuum or nitrogen sources, this method retains trace dissolved oxygen within the liquid solution, which can be overcome with longer polymerization times.

7. Incubate CLARITY Hydrogel at 37°C to complete polymerization of Solid Phase gel

    Recommended incubation times:
    A4B4P4: 3 hours (5 hours for Oil Method)
    A4B4P0: 3 hours (5 hours for Oil Method)
    A1B1P4: 4 hours (10 hours for Oil Method)

8. Remove Liquid Phase solution by pouring into an appropriate waste container.

9. (Optional) If sample is to be used for RNA studies, fix tissue with EDC solution at 37°C overnight as previously reported[4].

EDC Solution:

Chemical	Mass in 10 mL	Final Concentration
EDC	0.19 g	0.1M
ETT	0.13 g	0.1M
Methylmidizole Buffer (80 µl Methylmidizole in 10 mL H_2O)	80 µl	-

Supplemental Table 6: EDC Solution. This fixative helps preserve RNA under CLARITY.

10. (Optional) Cut tissue into thick slabs for facilitated clearing, staining, or imaging.

For thick (0.5-1 mm) section analysis, tissue handling can be facilitated if tissues are embedded within blocks of 2% agarose prior to sectioning with a vibratome or tissue block. Agarose maintains tissue rigid shape and flatness even under harsh conditions such as clearing solutions or high temperatures, but does not otherwise interfere with CLARITY processing or imaging.

11. Remove sample lipids using Clearing Solution. Samples are cleared in 4% SDS solution passively with shaking until clear.

Clearing Solution can be changed as infrequently as 1x per week.

Clearing Solution (pH to 8.5 with NaOH):

Chemical	Mass in 1L	Final Concentration
Sodium Tetraborate	40.24g	0.2M
Sodium Dodecyl Sulfate (SDS)	40g	4%
dH_2O	1L	-

Supplemental Table 7: Clearing Solution. 4% SDS in Sodium Borate buffer is an effective clearing solution.

Alternative Clearing Solution:

Chemical	Mass in 1L	Final Concentration
Sodium Dodecyl Sulfate (SDS)	40g	4%
1x PBS	-	-

Supplemental Table 8: Alternative Clearing Solution. 4% SDS in Phosphate buffer is also an effective clearing solution.

As previously reported[2,5], SDS concentration can be raised to 8%, which modestly improves clearing speed. We have found that an alternative clearing solution consisting of 4-8% SDS in 1x PBS is capable of clearing tissue at similar rates.

Clearing should be performed at the appropriate temperature. Prolonged exposure to temperatures higher than ~40°C will quench endogenous fluorescent proteins[7] so should not be used when preservation of endogenous fluors are desired. A4B4P0 gels will clear rapidly at elevated temperatures (60°C) but also lose protein quickly, so careful optimization of clearing temperature will be necessary depending on the molecular marker of interest. However, other gels including A1B1P4 and A4B4P4 should be cleared at elevated temperatures when possible for maximal speed.

For human tissues, clearing at elevated temperatures with A1B1P4 hydrogel is recommended for maximal clearing speed and protein retention.

Approximate Clearing Times (per 2 mm slice):

Tissue Type	A1B1P4, 37°C	A1B1P4, 60°C	A4B4P0, 37°C	Notes
Mouse Brain	7 days	2 days	2 days	
Mouse Muscle	10 days	3 days	2 days	Tendonous/cartilaginous areas may not clear fully and cause tissue warping.
Mouse Liver	-	-	1-2 weeks	A1B1P4 gels do not clear biliary trees fully
Mouse Kidney	1-2 weeks	6 days	5 days	
Mouse Pancreas	9 days	4 days	2 days	
Mouse Spleen	2-3 weeks	6 days	3-5 days	High hemoglobin content will prevent full transparency, which should not affect fluorescent imaging in far red range. May be eliminated through tissue decolorization[8,9]
Mouse Intestine	1-2 weeks	4 days	2-3 days	Feces will not clear
Mouse Heart	1-2 weeks	10 days	1 week	Heart chambers will fill with gel and rupture if not using Liquid Phase gel for polymerization
Mouse Lung	4-5 weeks	2 weeks	1 week	
Mouse Testis	2-3 weeks	10 days	2-3 days	
Mouse Ovary	1-2 weeks	3-5 days	2-3 days	
Mouse Bone	5-6 weeks	3-4 weeks	2-3 weeks	Tissue should be decalcified with EDTA or similar agent prior to clearing (or prior to hydrogel embedding)
Mouse Embryo (e11.5)	1-2 weeks	3-5 days	3-5 days	
Mouse Embryo (e13.5)	2-3 weeks	1 week	1 week	Cranial cavity may rupture if not using Liquid Phase gel for polymerization
Mouse Embryo (e15.5)	3-4 weeks	1-2 weeks	1-2 weeks	
Human Pancreas (Fetal)	3-4 weeks	2 weeks	-	
Human Pancreas (Juvenile/Adult)	3-4 weeks	2 weeks	-	A4B4P0 gels were not attempted with human samples due to risk of protein loss
Human Tumor Biopsy (Frozen)	2-3 weeks	1 week	-	

*Note, clearing of A4B4P0 tissue is not recommended at 60°C due to accelerated loss of proteins

Supplemental Table 9: Approximate Clearing Times. Empirical estimates for the duration for passive clearing in Clearing Solution of 2 mm tissue slices.

12. When tissue is clear, remove from SDS Clearing Solution and wash in PBST (1x PBS + 0.1% Triton-X) 3x, at least one hour each, and once overnight at room temperature.

    Clarity is most easily judged by observing a uniform translucency throughout the tissue. Partially cleared tissue will frequently have spots of greater opacity toward the center.

    Thoroughly washing the sample to remove trace amounts of SDS is critical. Residual SDS will precipitate when the sample is placed in refractive index matching solutions, causing opacity and tissue damage.

Molecular Labeling
In general, CLARITY is compatible with all existing forms of fluorescence-based molecular labeling, including antibody staining, in situ hybridization*, and small molecule dyes such as DAPI or Propidium Iodide for labeling nuclei. Autofluoresence of the tissue, particularly in short wavelength channels (i.e. 488nm), combined with nuclear labeling is sufficient to provide gross structural information analogous to Hematoxylin/Eosin staining.

Due to the nature of imaging modalities used with CLARITY, it is not compatible with non-fluorescent, colorimetric stains sometimes used in thin-section histopathology with bright field imaging, such as Crestyl Violet or Diaminobenzidine-Peroxidase, as these can create imaging artifacts while casting shadows through large image volumes. However, should users desire such stains, this can be overcome with the use of well-established fluorescent analogs, including NeuroTrace (ThermoFisher) and Tyramide Signal Amplification (TSA) (Perkin-Elmer) reagents.

*For detailed protocol information regarding nucleic acid labeling, see Sylwestrak et al., 2016[4].

1. Antibody Staining
   As with all antibody-based protocols, specific staining conditions will need to be optimized for individual probes, as each antibody has unique properties related to binding affinity, epitope specificity, and other biophysical properties that determine optimal staining conditions. We describe general best practices that have been effective for a large number of antibodies tested by our lab and others[1,3,5,10-12].

   Staining can be performed in PBST buffer at room temperature with shaking, in volumes sufficient to fully immerse the sample (typically 1-2 mL for tissue sections 2 mm or smaller), with thorough washing (at least 3x 1 hour each with one overnight wash) with PBST following both primary and secondary antibody incubations.

   Antibody concentration depends on the size of the tissue and abundance of the epitope. We have found that 1:200 dilution is sufficient for even moderately abundant markers (i.e. insulin in the pancreas or parvalbumin in the brain) but higher concentrations of 1:50 or more may be required for extremely abundant markers (such as neurofilament in the brain). For large samples, replenishment of additional antibody after several days of incubation may be beneficial.

Incubation time depends on gel type and size of sample.

Recommended minimum antibody incubation time (per 1 mm thickness):

- A4B4P4: 48 hours
- A1B1P4: 12 hours
- A4B4P0: 48 hours

Unlike with thin section histology, we have found there is no additional benefit to treating tissues with a blocking reagent prior to antibody staining, with the exception of when immunolabeling CLARITY samples derived from frozen clinical specimens.

2. Antibody Removal

After imaging, antibodies can be disassociated from their epitopes by incubation in Clearing Solution at 60°C[1,10]. As the antibodies are not fixed to the hydrogel backbone, the Clearing Solution will "clear" the antibody probes similar to other cleared macromolecules. While the precise incubation time required will depend heavily on antibody binding strength, a general guide is about 1 day of clearing per 500um of stained tissue. It is recommended that the user confirm the removal of antibodies by checking the tissue under an epifluorescent or confocal microscope for signs of fluorescence. If desired, the user can verify that primary antibodies are also removed by staining again with only secondary antibodies.

3. Subsequent rounds of Staining

Once antibodies are removed, the next round of staining can proceed exactly as indicated above (Step 1). Note that while every effort is made to minimize protein loss through overclearing, some is unavoidable; therefore it is recommended to prioritize detection of low-abundance signals in early rounds of staining, and high-abundance signals in later rounds.

4. Conjugated Antibodies

Our lab and others[1,3,13] have found success with the use of dye-conjugated antibodies, either commercially available (i.e. Alexa-conjugated anti-GFP, ThermoFisher A-31852) or conjugated in the lab using commercially available kits following the manufacturer's instructions (Alexa Fluor Antibody Labeling Kit – ThermoFisher A-20186).

Advantages of using conjugated antibodies include a reduced experiment duration (due to the need for only one antibody incubation rather than two) and a greater ability to multiplex (due both to the ability to simultaneously use primary antibodies from the same species without signal cross-contamination, and the ability to perform multi-round staining by quenching fluorescent signals from previous rounds rather than complete antibody removal.

Disadvantages of conjugated antibodies include the loss of signal amplification that occurs between primary antibody detection and secondary antibody detection, which can be crucial for the visualization of some low abundance markers.

Imaging

High-resolution and rapid imaging of CLARITY-processed tissue is discussed extensively in previous reports[1,3,14]. As all images in this report were generated with a commercially available confocal microscope (Olympus FV-1100) with a standard objective lens (10x water immersion, 0.6NA, 3 mm WD, Olympus), we summarize our procedure below.

1. Place sample in Refractive Index Matching reagent for 1-48 hours (depending on sample size).

   Many commercial and home-made solutions for refractive index matching have been reported, including FocusClear, Glycerol, Histodenz, ScaleA2, TDE, and RapiClear[1,5,6,10,13,15]. In this study, we use RapiClear as we have found it provides great transparency, is cost-effective, and can be reused on multiple samples.

   As users select the reagent most appropriate for them, several key considerations include:

   a. Cost
   Though commercial reagents can be expensive (for example, FocusClear), others are inexpensive (Histodenz, RapiClear) and comparable in price to homemade solutions.

   b. Sample swelling
   Several reagents, such as Glycerol, are known to significantly swell or shrink the tissue from its original dimensions, which could be advantageous (for example, for resolving fine structural features[16]) or disadvantageous (increased imaging time), depending on specific applications. The reagent combinations used in this study (A1B1P4 gel with RapiClear) do not significantly change tissue dimensions.

   c. Ability to titrate Refractive Index
   Commercial reagents are optimized for tissues with refractive index of 1.45. However, the refractive index of a sample may be higher or lower depending on properties such as macromolecular content. For example, calcified bones have a refractive index of approximately 1.53[17-19]. Homemade reagents are easily tuned to have optimal refractive indices for any tissue specimen, as described previously for Histodenz[5], TDE[6], and diatrozic acid/iodixanol[10]. It is recommended that users check the final refractive index of their solutions using a commercially available refractometer.

2. Mount sample in imaging chamber constructed from a glass slide, clay, and a coverslip or glass dish, as previously described[1,3,4].

3. Fill imaging chamber with refractive index solution, remove bubbles and seal with Kwik-Sil (World Precision Instruments, #KWIK-SIL).

4. Obtain confocal images following manufacturer's instructions.

5. Analyze images using appropriate tools such as free analysis software (ImageJ), commercial 3D image analysis software (Imaris, Bitplane), or custom image processing software, depending on application.

CLARITY Extensions

Biphasic CLARITY is a modular approach that may be extended and combined with innovations from other volumetric histology methods with minimal re-engineering. Decolorization reagents from CUBIC may be used to remove heme elements from CLARITY tissues[9], higher temperatures may be used to increase reaction kinetics[7], antibody penetration may be improved with the use of binding and non-binding buffers[10], and refractive index matching may be accomplished using a wide variety of affordable, commercially available reagents and home-made mixtures, including FocusClear, RapiClear, glycerol, Histodenz, 2,2'-thiodiethanol, CUBIC-2, or diatrizoic acid/iodixanol[1,5,8,10,13,20]. Additionally, end-users can select advanced components, such as custom perfusion chambers and light sheet microscopes[2,3], to add onto a simple CLARITY framework in a modular manner depending on specific application.

Supplementary References:

1 Chung, K. *et al.* Structural and molecular interrogation of intact biological systems. *Nature* **497**, 332-337, doi:10.1038/nature12107 (2013).

2 Treweek, J. B. *et al.* Whole-body tissue stabilization and selective extractions via tissue-hydrogel hybrids for high-resolution intact circuit mapping and phenotyping. *Nature protocols* **10**, 1860-1896, doi:10.1038/nprot.2015.122 (2015).

3 Tomer, R., Ye, L., Hsueh, B. & Deisseroth, K. Advanced CLARITY for rapid and high-resolution imaging of intact tissues. *Nature protocols* **9**, 1682-1697, doi:10.1038/nprot.2014.123 (2014).

4 Sylwestrak, E. L., Rajasethupathy, P., Wright, M. A., Jaffe, A. & Deisseroth, K. Multiplexed Intact-Tissue Transcriptional Analysis at Cellular Resolution. *Cell* **164**, 792-804, doi:10.1016/j.cell.2016.01.038 (2016).

5 Yang, B. *et al.* Single-cell phenotyping within transparent intact tissue through whole-body clearing. *Cell* **158**, 945-958, doi:10.1016/j.cell.2014.07.017 (2014).

6 Zheng, H. & Rinaman, L. Simplified CLARITY for visualizing immunofluorescence labeling in the developing rat brain. *Brain structure & function* **221**, 2375-2383, doi:10.1007/s00429-015-1020-0 (2016).

7 Yu, T. T., Qi, Y. S., Gong, H., Luo, Q. M. & Zhu, D. Optimized optical clearing method for imaging central nervous system. *Proc Spie* **9305**, doi:Artn 930527 10.1117/12.2078736 (2015).

8 Susaki, E. A. *et al.* Whole-brain imaging with single-cell resolution using chemical cocktails and computational analysis. *Cell* **157**, 726-739, doi:10.1016/j.cell.2014.03.042 (2014).

9 Tainaka, K. *et al.* Whole-body imaging with single-cell resolution by tissue decolorization. *Cell* **159**, 911-924, doi:10.1016/j.cell.2014.10.034 (2014).

10 Murray, E. *et al.* Simple, Scalable Proteomic Imaging for High-Dimensional Profiling of Intact Systems. *Cell* **163**, 1500-1514, doi:10.1016/j.cell.2015.11.025 (2015).

11 Zhang, M. D. *et al.* Neuronal calcium-binding proteins 1/2 localize to dorsal root ganglia and excitatory spinal neurons and are regulated by nerve injury. *Proceedings of the National Academy of Sciences of the United States of America* **111**, E1149-1158, doi:10.1073/pnas.1402318111 (2014).

12 Font-Burgada, J. *et al.* Hybrid Periportal Hepatocytes Regenerate the Injured Liver without Giving Rise to Cancer. *Cell* **162**, 766-779, doi:10.1016/j.cell.2015.07.026 (2015).

13 Ye, L. *et al.* Wiring and Molecular Features of Prefrontal Ensembles Representing Distinct Experiences. *Cell*, doi:10.1016/j.cell.2016.05.010 (2016).

14 Richardson, D. S. & Lichtman, J. W. Clarifying Tissue Clearing. *Cell* **162**, 246-257, doi:10.1016/j.cell.2015.06.067 (2015).

15 Liu, A. K. *et al.* Bringing CLARITY to the human brain: visualisation of Lewy pathology in three-dimensions. *Neuropathology and applied neurobiology*, doi:10.1111/nan.12293 (2015).

16 Chen, F., Tillberg, P. W. & Boyden, E. S. Optical imaging. Expansion microscopy. *Science* **347**, 543-548, doi:10.1126/science.1260088 (2015).

17 Acerbo, A. S., Carr, G. L., Judex, S. & Miller, L. M. Imaging the material properties of bone specimens using reflection-based infrared microspectroscopy. *Analytical chemistry* **84**, 3607-3613, doi:10.1021/ac203375d (2012).

18 Calve, S., Ready, A., Huppenbauer, C., Main, R. & Neu, C. P. Optical clearing in dense connective tissues to visualize cellular connectivity in situ. *PloS one* **10**, e0116662, doi:10.1371/journal.pone.0116662 (2015).

19 Pifferi, A. *et al.* Optical biopsy of bone tissue: a step toward the diagnosis of bone pathologies. *Journal of biomedical optics* **9**, 474-480, doi:10.1117/1.1691029 (2004).

20 Costantini, I. *et al.* A versatile clearing agent for multi-modal brain imaging. *Scientific reports* **5** (2015).
